# *Meta*‐Connected *Oligo*‐Azobenzenes Outperform Their *Para* Counterparts

**DOI:** 10.1002/cphc.202400799

**Published:** 2024-11-29

**Authors:** Nils Oberhof, Leon Kambiz Paschai Darian, Andreas Dreuw

**Affiliations:** ^1^ Interdisciplinary Center for Scientific Computing Heidelberg University Im Neuenheimer Feld 205 69120 Heidelberg Germany

**Keywords:** energy conversion, energy storage, photochemistry, quantum chemistry, azo compounds

## Abstract

Systems with multiple photoswitchable units in one molecule have attracted considerable attention in the past years as they are useful for a broad variety of possible applications. Especially, linked azobenzenes sharing one benzene ring are of high interest since their direct linkage introduces an additional photoswitchable unit at only small increase in molecular weight. In this spirit, linear *oligo*‐azobenzenes had been synthesized, though their photochemical properties have only been investigated for short chain lengths. In this study, we use (time‐dependent) density functional methodology for the evaluation of the excitations of *meta*‐ and *para*‐connected *oligo*‐azobenzenes to predict their switching ability. It becomes apparent, that the *meta* connection pattern enables each azobenzene subunit to act as an individual switchable unit, whereas they are strongly coupled and loose their individuality in *para* connection. Therefore, *meta*‐*oligo*‐azobenzenes are ideal candidates for future studies of azobenzene‐based functional polymers, while *para*‐*oligo*‐azobenzenes are not.

## Graphical abstract

Meta switches better. Our computational investigation of quasi‐linear *oligo*‐azobenzenes with *para*‐ and *meta*‐connectivity reveals *meta* to result in oligomers with individually photoswitchable azobenzene subunits. In contrast, the excitonic delocalization in *para* lets the azobenzene subunits lose their individuality resulting in vanishing photoswitchability with oligomer length.

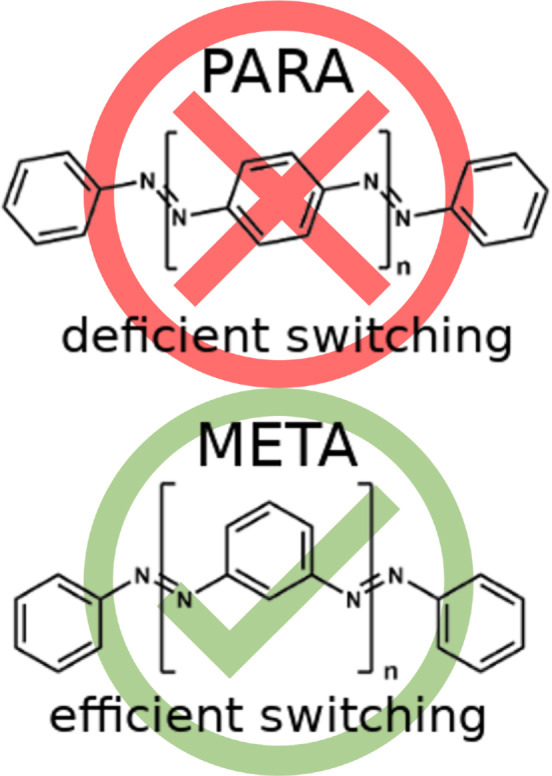

## Introduction

In the past, azobenzene (AB) and its derivatives have become prototypical examples of photoswitches with many different possible applications in nanotechnology,[^1^, ^2^, ^3^] host‐guest chemistry,^[4]^ molecular machines,^[5]^ solar energy storage,[^6^, ^7^, ^8^] photoresponsive materials^[9]^ and other areas of research.[^10^, ^11^] The most obvious reason for its broad applicability is the large geometrical change upon its isomerization from (*E*)‐ to (*Z*)‐AB, which, however, does not limit its ability to switch even in solid‐state environments or polymeric matrices.[^12^, ^13^] The photoisomerization of AB proceeds on the PES of the lowest excited state *S*_1_, which can be populated either via direct excitation or via internal conversion from higher states. In (*E*)‐isomers the direct population of *S*_1_ is less likely due to the low oscillator strength than in (*Z*)‐isomers.^[14]^ E‐ to Z switching of azobenzene is usually accompanied with a substantial amount of energy of approximately 49 kJ/mol that can be stored in the metastable (*Z*)‐state of azobenzene derivatives with a reasonable half‐life time making them generally attractive as molecular solar thermal energy storage (MOST) systems.^[6]^ Several combinations of azobenzenes with (other) photoswitches have been investigated previously, where different linkage strategies have been applied, such as perpendicular orientation or aliphatic linkers.[^15^, ^16^, ^17^] Direct combination of two azophenyl groups attached to one central benzene ring, however, gives an atom‐economical alternative to linking two full ABs which has been investigated for bis‐azobenzenes.[^18^, ^19^, ^20^, ^21^]

Additionally investigations on “star‐like”‐trisazobenzene photoswitches have been carried out, in which three azophenyl groups are attached to one central benzene ring.[^22^, ^23^] In MOST applications, the energy storage density of a specific system, i. e. the ratio between stored energy and molecular weight, is one of the most important applicability criteria. In this context, the direct connection of azophenyl units to a single central benzene ring constitutes a significant advantage compared to the combination of two full AB subunits via an additional linker, since the added mass per energy storage unit is much smaller. In other words, the energy storage density is increased by means of this strategy. When combining multiple azobenzenes in this way, their connectivity has a drastic influence on the switching behavior of the individual azobenzene subunits. In the bis‐azobenzene systems *ortho*‐connectivity leads to strong intramolecular excitonic coupling between the individual AB subunits that results in fast relaxation dynamics and therefore no accumulation of (*Z*)‐isomers. *Para*‐connectivity on the contrary leads to strong delocalization of the *π*‐system resulting in red‐shifted absorption bands and strongly reduced switching quantum yields. In contrast, the *meta*‐connected bis‐AB exhibits quasi‐independent *π*‐systems of the individual AB subunits, enabling independent and efficient switching as in isolated AB.[^18^, ^19^, ^20^, ^21^] This concept has been expanded to tris‐*meta*‐azobenzenes sharing a central benzene ring to result in three independent photoswitches in one single molecule.^[22]^ Following the same structural concept of *meta*‐connectivity, triptycene‐azobenzene derivatives as well as a combination between AB and spiropyran have shown individual switching behavior.[^24^, ^25^] Derivatives of aforementioned bis‐azobenzene systems have also been incorporated in polymeric materials, where they show the previously predicted behavior comparable to the single molecules with an individual adressability of the different azobenzene groups in *meta*‐connectivity while the *para*‐connected bis‐azobenzene can only be addressed as one photoswitch.[^26^, ^27^, ^28^]

**Figure 1:**
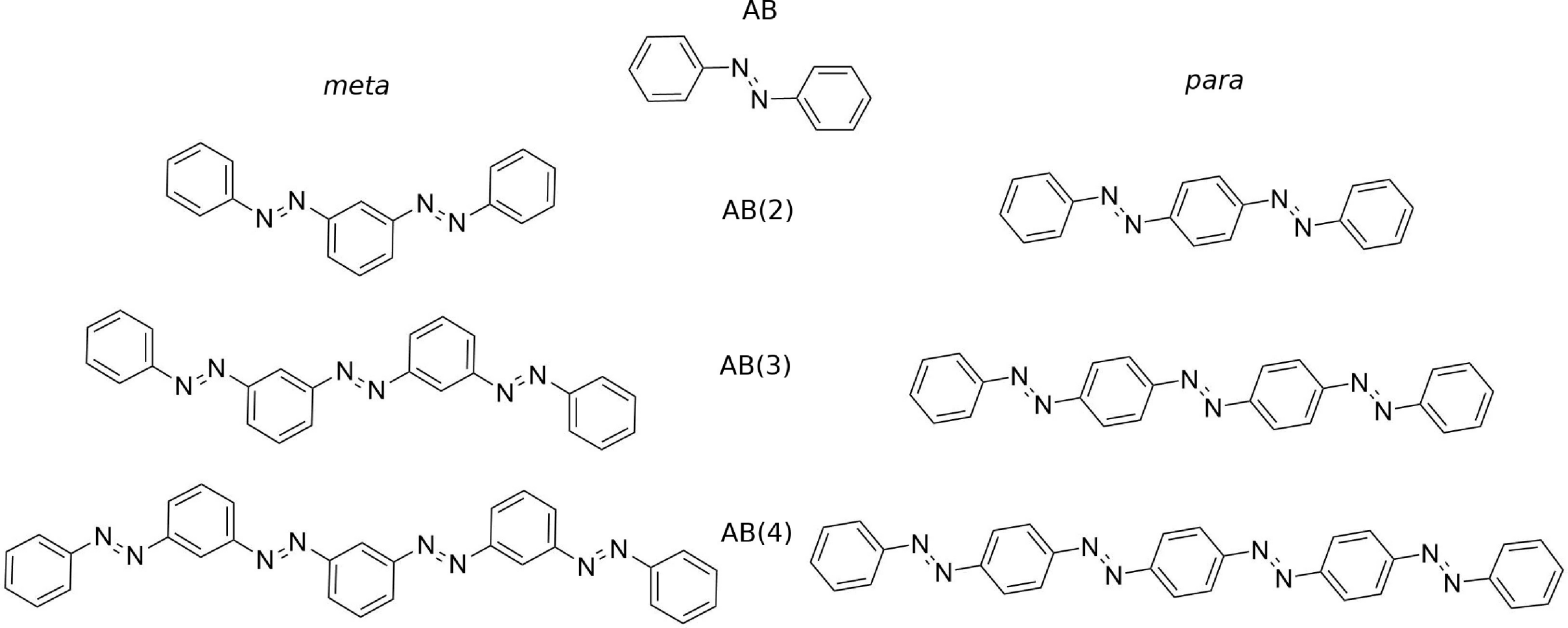
Molecular structures of the all‐(*E*) *meta*‐ and *para*‐connected AB(*n*) oligomers with *n*=1–4.

In the 1930s and 40s, some linear *oligo*‐azobenzenes (see Figure 1) have been synthesized and in the 50s first absorption spectra were measured.[^29^, ^30^, ^31^] The two lowest‐lying absorption bands show a strong redshift for those oligomers, which exhibit *para*‐connectivity. In contrast, for the *meta*‐connected oligomer the absorption spectrum is practically identical to the AB monomer, but with a strongly increased absorption cross section. Despite their potential applicability in, for example, photo‐responsive materials or energy storage applications, further investigations of their electronic structure and molecular properties, such as photoswitching or excitonic coupling, have not been conducted except for linearly coupled bis‐azobenzenes.[^18^, ^19^, ^20^, ^21^]

A detailed theoretical investigation of the electronic structure of these linear *oligo*‐azobenzenes is critical in order to gain further insights into their properties, eventually allowing for derivation of structure‐function relationships as guidelines for further improvement. In this work, using (time‐dependent) density functional theory for the excited states of the *oligo*‐azobenzenes, absorption spectra have been simulated, detachment and attachment densities computed, and exciton properties used to understand the differences in their electronic structure. We aim at the prediction of the fundamental ability of switching without detailed investigation of the details of the exact mechanism. Thereby, the previously established, so‐called, “*meta*‐rule” is substantiated by demonstrating its vailidity also for the linear *oligo*‐azobenzenes. In fact, we predict individual photoswitching of the AB subunits in the *meta*‐*oligo*‐azobenzenes (*meta*‐AB(*n*)s, where *n* describes the number of AB units). On the other hand, the *para*‐*oligo*‐azobenzenes (*para*‐AB(*n*)s) are predicted to loose their photoswitchability with increasing length.

## Results and Discussion

### Photochemical Properties of All‐(*E*) *Oligo*‐Azobenzenes

For the investigation of directly linked *oligo*‐azobenzene derivatives, in which the benzene rings are shared between two azobenzene subunits, except the terminal ones, the *para*‐ and *meta*‐connected AB(*n*) oligomers with a length of up to four AB (*n*=4) subunits were investigated, which are displayed in Figure 1. Their optimized gas‐phase structures are planar and they possess a clear molecular point group symmetry: the all‐(*E*) *para*‐AB(*n*)s exhibit *C*_2*h*_ symmetry independent of *n*, while the all‐(*E*) *meta*‐AB(*n*)s possess *C*_2*v*_ symmetry for even *n* and *C*_2*h*_ symmetry for odd *n*. All calculations, however, were performed without imposing their molecular point group symmetry to avoid symmetry‐induced delocalization of the excited electronic states, which we will discuss later. This is important because we utilize the degree of delocalization of the vertical excited states as proxy to predict the photoswitching efficiency. Although it is usually not sufficient to investigate vertical excited states to predict dynamic properties like photoswitching efficiency, we have proven previously for the *ortho‐*, *meta‐* and *para‐*bisazobenzene in a joint experimental and theoretical study that the degree of delocalization of the vertical excited states indeed correlates with the quantum yield of photoswitching.^[20]^ Due to the very close structural relation of the studied systems here, the degree of delocalization of the vertical excited states can be applied to predict the qualitative and relative efficiency of photoswitching. The concept of delocalization has already been successfully applied to bis‐ and tris‐azobenzenes,[^18^, ^19^, ^22^] as well as triptycene‐linked ABs.^[24]^ For quantitative predictions, of course, more elaborate quantum chemical investigations of the isomerization pathways and subsequent dynamics simulations are mandatory.

### All‐(*E*) *para*‐AB(*n*) Oligomers

**Figure 2:**
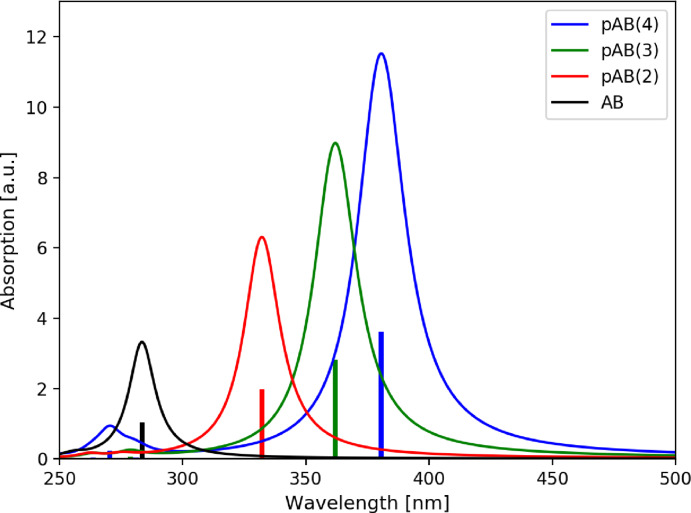
Simulated absorption spectra of the all‐(*E*) *para*‐AB(*n*)s with *n*=1–4. The calculated stick spectra (TDDFT/CAM‐B3LYP) have been convoluted with a Lorentzian function with a full width at half maximum (FWHM) of 0.2 eV.

First we consider the *para*‐connected AB(*n*) oligomers. Table 1 lists the energetically lowest lying singlet transitions, which originate from *n*→π* and π→π* excitations, which are relevant for the discussion of the photochemical and spectroscopic properties. Upon addition of each AB subunit another *n*→π* excitation, which in AB promotes the ultra‐fast E→Z photoswitching process, occurs at low energies within the system and all *n*→π* excitations become more red‐shifted with each additional unit. Similarly, for the π→π* excitations of the oligomers, of course, one additional low‐lying π→π* excitation arises with each added AB subunit. However, due to the strong coupling between them the additional states are pushed to the higher energy region and only the single energetically lowest one is obtained in the relevant energy region within and close to the visible spectral range. It experiences an even stronger redshift than the *n*→π* excitations, and its oscillator strength grows with every additional AB subunit (see Figure 2). This is in agreement with the previous experimental observation.^[31]^

**Table 1:** Vertical excitation energies (in eV) and oscillator strengths in parenthesis (in a.u.) of the relevant excited states of *para*‐AB(*n*)s at the TDDFT/CAM‐B3LYP level of theory.

Excitation	AB	*para*‐AB(2)	*para*‐AB(3)	*para*‐AB(4)
*n*→π*	2.83 (0.00)	2.74 (0.00)	2.69 (0.00)	2.67 (0.00)
*n*→π*		2.77 (0.00)	2.74 (0.00)	2.70 (0.00)
*n*→π*			2.75 (0.00)	2.75 (0.00)
*n*→π*				2.75 (0.00)
*n*→π*	4.37 (1.04)	3.73 (1.98)	3.43 (2.82)	3.26 (3.62)

The continuous decrease of the excitation energy of the energetically lowest excited states with the number of added AB subunits gives a clear indication of the delocalization of the *n*→π* and π→π* excitations. This already gives a strong hint that each AB unit can most probably not be switched individually, as it has been previously found in the investigation of the bis‐azobenzenes.[^20^, ^21^]

**Figure 3:**
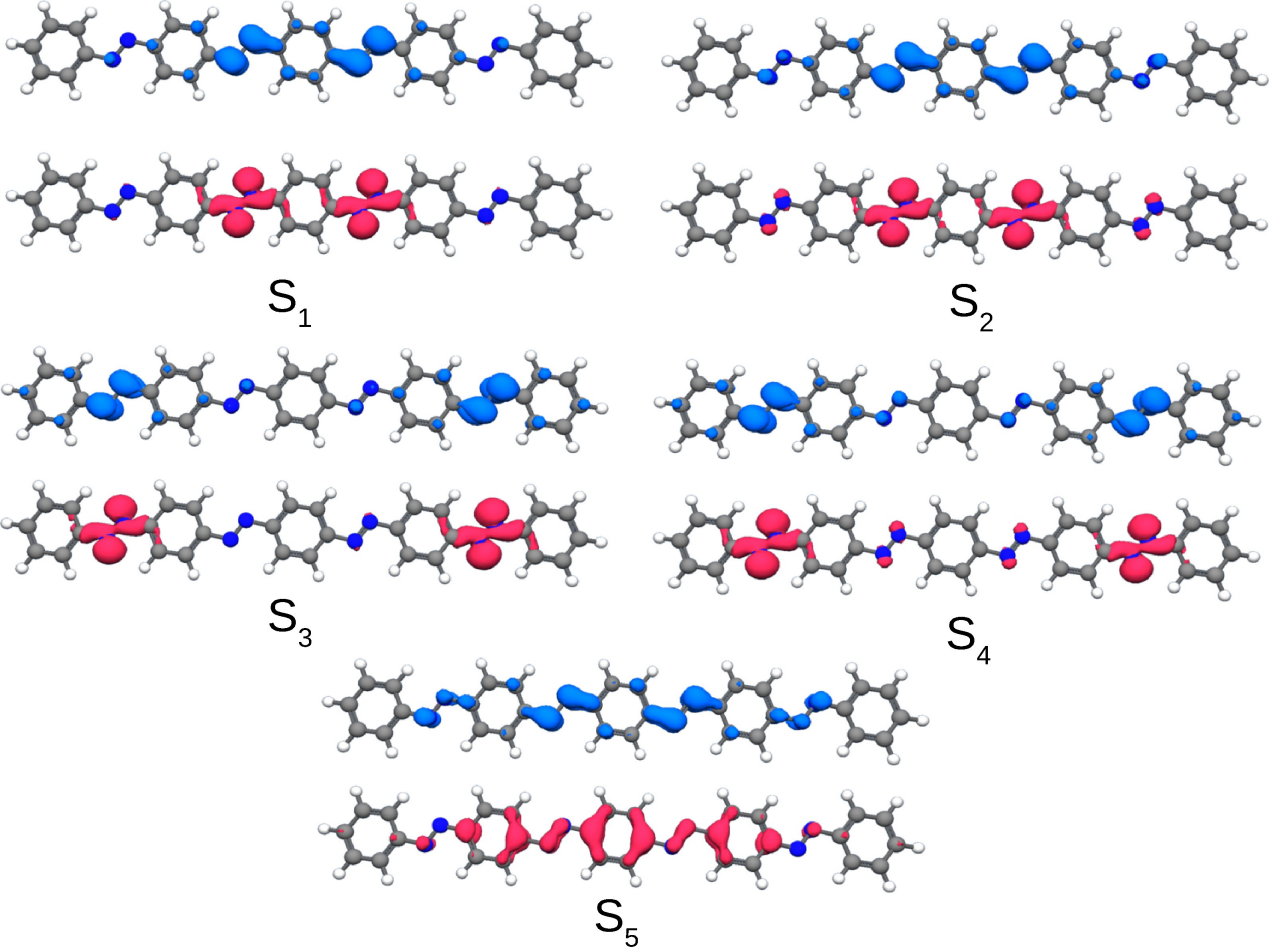
Detachment (red) and attachment (blue) densities of the first five singlet excited states of *para*‐AB(4) (isovalue 0.002, TDDFT/CAM‐B3LYP).

The latter finding can be further substantiated by inspection of the detachment and attachment densities (see Figure 3). These densities allow for the interpretation of electronic excited states that are composed of several orbital pairs, since the contribution of all participating orbitals is captured. The detachment density corresponds to the electronic density that is removed upon excitation, while the attachment density amounts to the electronic density readded upon the excitation.^[32]^ As a representative example, the lowest‐lying excitations for *para*‐AB(4) are discussed. The same findings however hold true also for the *para*‐AB(*n*)s with shorter chain lengths, i. e. *n*=1–3. Within the *para*‐AB(*n*), the energetically lowest π→π* excitation is fully delocalized over the whole *π*‐system, which can be clearly seen for the corresponding detachment and attachment densities of this state. Even the *n*→π* excitations show a strong delocalization over the (whole) AB(*n*) oligomers. These delocalized excitations contrast the local character of individually switching AB subunits. This suggests a reduced switching ability.

Since the inspected detachment and attachment densities correspond to the probabilities to find the corresponding hole and particle of the exciton in the *para*‐AB(4), and not strictly to the spatial localization of the excitation, we explicitly also computed the physical properties of the excitons, in particular, the exciton size of the lowest‐lying π→π* transition. As can be see in Table 2, the spatial size of the exciton keeps increasing significantly with each added AB unit from 3.9 to 5.8 Å from AB(1) to AB(4), confirming again the increasing spatial delocalization of the exciton. Since the vertical excitation energy decreases and the exciton becomes even further distributed over a larger spatial region of the oligomer with increasing *n*, we assume a strongly decreasing quantum yield for E→Z photoisomerization, which is a spatially localized event.

**Table 2:** Exciton size (in Å) of the first π→π* excitation of the *para* and *meta*‐AB(*n*) series (TDDFT/CAM‐B3LYP).

d_*exc*_ [Å]	AB	AB(2)	AB(3)	AB(4)
*para*	3.90	4.85	5.45	5.81
*meta*	3.90	4.10	4.05	4.05

A reduced switching efficiency can also be rationalized by invoking a simplified molecular orbital‐based bond order argument. Upon excitation of an electron from a *π* to a *π* ^*^ MO, the formal order of the *π*‐bonds is reduced in the excited state depending on the number of conjugated double bonds. For a single double bond, as in AB(1), the promoted electron reduces the bond order (BO) of the central N=N double bond from *BO*=2 (two *σ*‐bonding electrons and two *π*‐bonding electrons yielding two covalent bonds) to *BO*=1 by excitation of one *π* electron to the *π* ^*^ anti‐bonding orbital (two *σ*‐bonding electrons, one *π*‐bonding and one *π*‐anti‐bonding electron yield one covalent bond). For two conjugated double bonds with delocalized orbitals, the promotion of one electron does not lead to a formal reduction of the bond order of one double bond from *BO*=2 to *BO*=1 and to the possibility to rotate freely around the remaining single bond, but instead only to a reduction of the isolated double bonds to *BO*=1.5. This can be transferred to *para*‐AB(2), since the delocalized MOs lead to a similar situation. A total of four bonding *σ* and four bonding *π* electrons account for an average *BO*=2 for each of the individual azobonds of the AB units, which, however are only weakened to *BO*=1.5 upon excitation of a single *π* electron to the *π* ^*^ orbital delocalized over the whole system. Following this argument, this trend continues for *para*‐AB(3) (average BO in the energetically lowest π→π* excitation is formally 1.66) and for *para*‐AB(4) (average BO in the excited state of 1.75). Therefore, also from this oversimplified point of view, a reduced photoisomerization ability of the *para*‐AB(*n*) oligomers with increasing number of AB units is expected.

### All‐(*E*) *Meta*‐AB(*n*) Oligomers

For the *meta*‐connected AB(*n*) oligomers, the same systematic series of derivatives from *meta*‐AB(2) to *meta*‐AB(4) (see Figure 1) was investigated. Table 3 lists the energetically lowest‐lying singlet excited states, which also in this case originate from *n*→π* and π→π* transitions. For each added AB unit one additional *n*→π* transition occurs in the low‐energy region. In contrast to the *para*‐connected oligomers, the *meta*‐connected AB(*n*)s also acquire an additional π→π* excited state per AB unit in the relevant spectral region. Consequently four energetically low‐lying *n*→π* and four π→π* excitations are present in *meta*‐AB(4) within the relevant energy range. It is remarkable, that the excitation energies and oscillator strengths of the *n*→π* excitations barely change with increasing number of AB units added. This already hints at an independent behavior of these excitations and possibly also of each individual azobenzene subunit.

**Table 3:** Vertical excitation energies (in eV) and oscillator strengths in parenthesis (in a.u.) of the relevant excited states of *meta*‐AB(*n*)s at the TDDFT/CAM‐B3LYP level of theory.

Transition	AB	*meta*‐AB(2)	*meta*‐AB(3)	*meta*‐AB(4)
*n*→π*	2.83 (0.00)	2.82 (0.00)	2.82 (0.00)	2.81 (0.00)
*n*→π*		2.82 (0.00)	2.82 (0.00)	2.82 (0.00)
*n*→π*			2.82 (0.00)	2.82 (0.00)
*n*→π*				2.82 (0.00)
π→π*	4.37 (1.04)	4.23 (1.60)	4.16 (2.61)	4.11 (3.63)
π→π*		4.32 (0.26)	4.29 (0.01)	4.26 (0.00)
π→π*			4.30 (0.40)	4.28 (0.35)
π→π*				4.28 (0.23)

**Figure 4:**
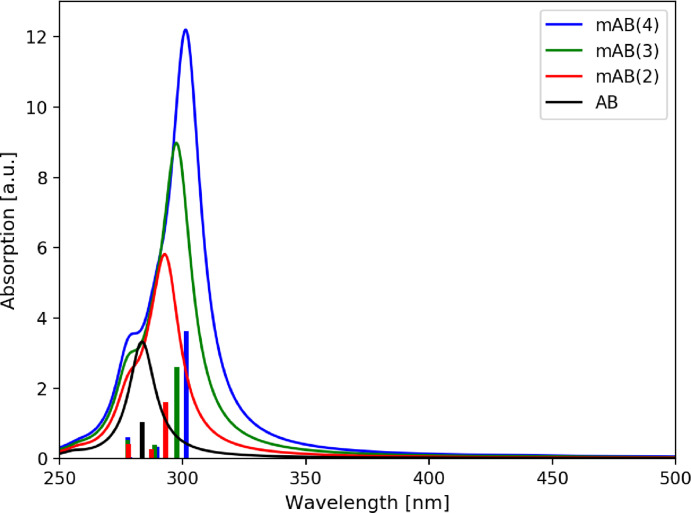
Calculated absorption stick spectra (TDDFT/CAM‐B3LYP) of the all‐(*E*) *meta*‐AB(*n*)s convoluted with a Lorentzian function with 0.2 eV FWHM.

**Figure 5:**
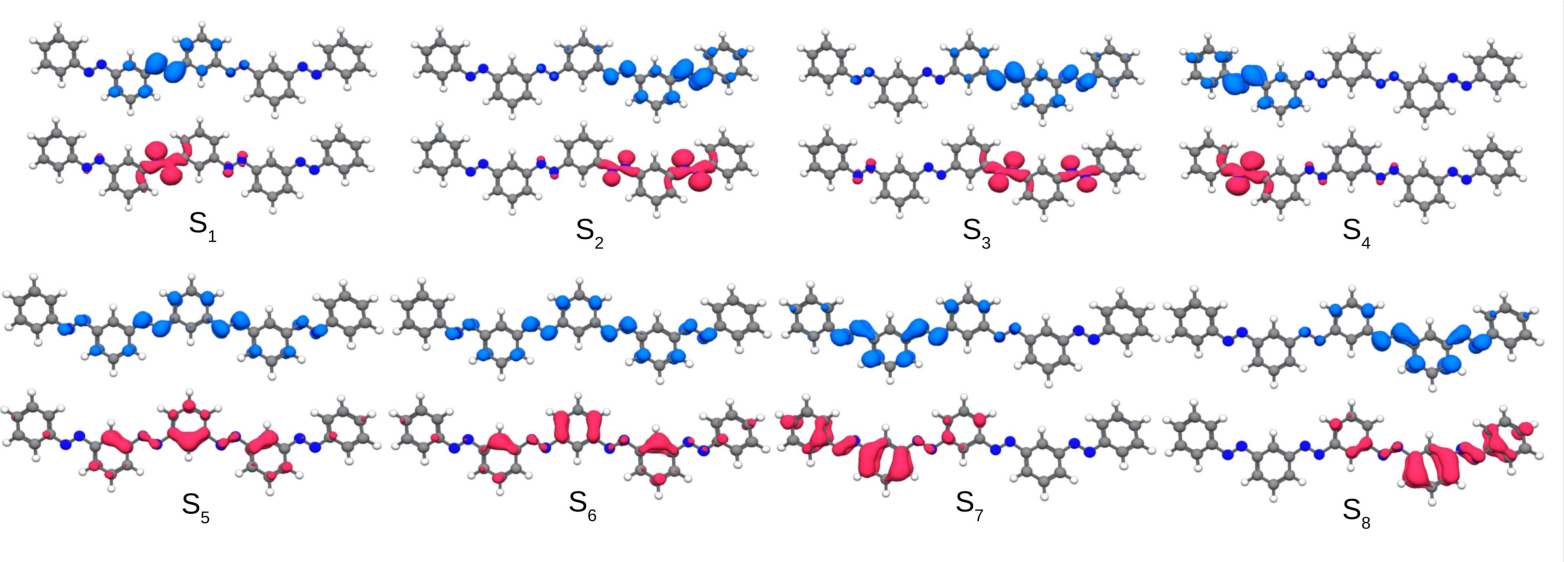
Detachment (red) and attachment (blue) densities of the first eight excited singlet states of *meta*‐AB(4) (isovalue 0.002, TDDFT/CAM‐B3LYP).

A slightly more complicated situation is encountered for the π→π* transitions of the *meta*‐AB(*n*) oligomers. As can be seen in Table 3, also the energy of the lowest π→π* transition hardly changes upon addition of additional AB subunits, only from 4.37 to 4.11 eV compared to the *para*‐AB(*n*) oligomers, in which the excitation energy reduces to 3.26 eV. In the simulated absorption spectra (Figure 4), the main absorption peak experiences thus only a small redshift, while significantly gaining oscillator strength when going from AB(1) to *meta*‐AB(4). Obviously, the π→π* transitions of the AB subunits are much weaker coupled and keep much of their individuality. Therefore, their interaction can be approximately related to a simple excitonic coupling scheme, which leads to the following pattern for the *meta*‐AB(*n*) oligomers. In *meta*‐AB(2) a small splitting into one stronger stabilized transition with high oscillator strength and one less stabilized transition with low oscillator strength is observed. For *meta*‐AB(3) they split into one stabilized transition with strong oscillator strength, and two less stabilized transitions with one transition having no and one exhibiting small oscillator strength. In *meta*‐AB(4) this effect is even more pronounced. Here, one transition is relatively strongly stabilized and exhibits very strong oscillator strength, and three are less stabilized with vanishing oscillator strengths. However, the calculated spectral shift is relatively small in all three cases, and the simulated spectra are very reminiscent of appropriate multiples of the unperturbed (*E*)‐AB spectrum. For all *meta*‐*oligo*‐azo‐benzenes discussed above the oscillator strength of the coupled π→π* transitions add up to approximately the n‐fold of that of the single AB π→π* transition. This finding is in line with the measured experimental spectra of *meta*‐*oligo*‐azobenzenes recorded in the 1950s.^[31]^ This strongly suggests the AB subunits of the *meta*‐AB(*n*) oligomers to keep their individual properties including their individual photoswitchability. The locality of these relevant excitations of the *meta*‐AB(*n*) oligomers becomes fully apparent by an analysis of the corresponding detachment and attachment densities. As a representative example the lowest‐lying excitations are discussed again for the *meta*‐AB(4) oligomer (see Figure 5), since identical findings apply also to the smaller oligomers. The first four excited states, corresponding exclusively to *n*→π* transitions, are all well localized on isolated AB subunits (Figure 5). For the π→π* excitations this is slightly less obvious but can be rationalized. The partial delocalization of the detachment and attachment densities originates from the excitonic coupling of the four isolated π→π* transitions, localized on the N=N bonds, transforming in the present quasi *C*_2*v*_ symmetry. However, as seen above, the densities reflect only the probability to find the corresponding hole or particle at a certain point in the oligomer, and not directly its spatial delocalization. To quantify the initial spatial distribution of the excitation energy in the oligomers, again, the size of the exciton of the lowest‐lying π→π* excitation has been calculated for the *meta*‐*oligo*‐azobenzenes. Upon introduction of the second AB unit the exciton size increases slightly in *meta*‐AB(2) from 3.9 to 4.1 Å, however, much less than in *para*‐AB(2), where the exciton has already a size of 4.85 Å. Upon further introduction of meta‐connected AB units, the exciton size remains virtually the same in *meta*‐AB(3) and *meta*‐AB(4) even with a negligible decrease at 4.05 Å (Table 2). This strongly suggests that the electronic structure of the individual AB subunits does not significantly change in the *meta*‐AB(*n*) oligomers, as is reflected in unchanged absorption characteristics as well as in their localization at individual AB subunits. This strongly suggests that the individual AB subunits keep their capability as photoswitch.

*Meta*‐connected AB oligomers are thus photoswitchable systems with significant quantum yields similar to that of isolated AB, while *para*‐connected oligomers will hardly be photoswitchable at all. In principle, *meta*‐AB(*n*) oligomers can also be individually E→Z switched, when their π→π* excitation can be individually optically addressed. This can in principle be achieved by selective chemical substitution of isolated AB subunits shifting their absorption peak away from the others, similar in spirit to the idea proposed in Ref. [22].

### Spectroscopic Signatures of *E* → *Z* Switched AB(*n*) Oligomers

For guidance of future spectroscopic investigations and experimental photochemical investigations of the linear AB(*n*) oligomers, the excited electronic states of different E→Z switched AB(*n*) oligomers have been calculated and their absorption spectra simulated in strict analogy to those presented previously for the all‐(*E*) isomers. This will help to identify specific spectral signatures of singly, doubly, triply and up to quadruply E→Z switched AB(*n*) isomers with *n*=1–4. The applied nomenclature will be the following demonstrated for AB(4) oligomers: (*ZEEE*)‐AB(4) and (*EZEE*)‐AB(4) refer to AB(4) isomers in which only the first or only the second AB subunit is in (*Z*) configuration. For symmetry reasons (*ZEEE*)‐AB(4) and (*EEEZ)*‐AB(4) are identical, as well as (*EZEE*)‐AB(4) and (*EEZE*)‐AB(4). In strict analogy, (*ZZEE*)‐AB(4) and (*ZEZE*)‐AB(4) as well as (*EZZE*)‐AB(4) refer to three different doubly switched AB(4) oligomers. The nomenclature for triply switched isomers follows the same logic. Note that (*Z*)‐azobenzene and oligomers containing at least a single E→Z switched AB subunit possess helical chirality, which leads to the existence of enantiomers and diastereomers depending on the number of AB subunits and their individual helicity. For simplicity, *M* chirality was chosen for all (*Z*)‐conformations in our computations because the specific helical chirality has no significant influence on the linear absorption spectra of the oligomers. This is of course different when electronic circular dichroism is studied, specifically probing chirality, however, this goes beyond the scope of this manuscript. In the following, we will discuss the linear absorption spectra of the E→Z switched *para*‐ and *meta*‐connected AB(4) oligomers as examples, since the findings are directly transferable also to the smaller oligomers.

**Figure 6:**
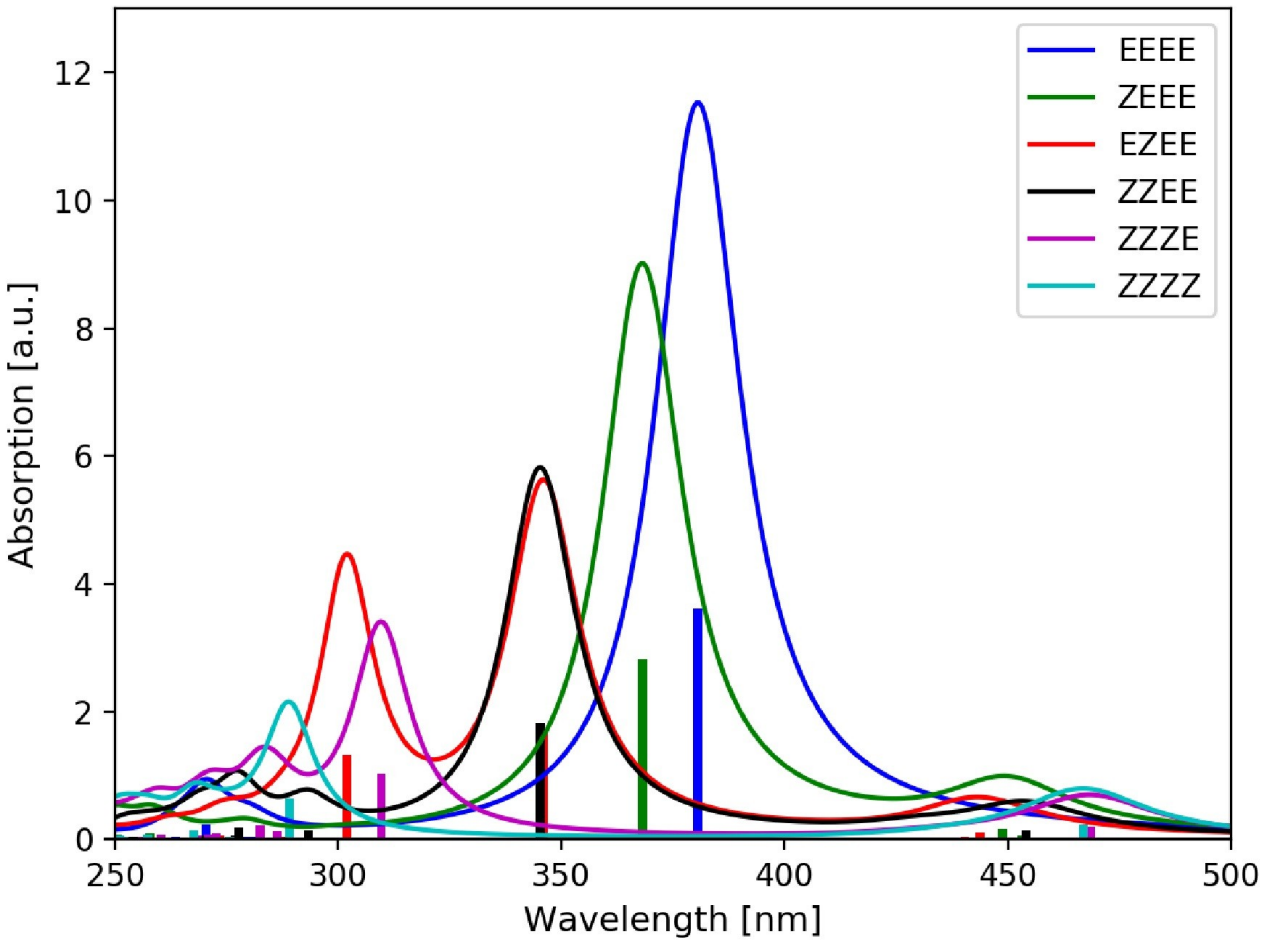
Simulated absorption spectra (TDDFT/CAM‐B3LYP) of *para*‐AB(4)s with varying numbers of (*Z*)‐AB units. All (*Z*)‐isomers considered here are of *M* chirality. The stick spectra were convoluted with a Lorentzian function of 0.2 eV FWHM.

Starting our computational investigation with *para*‐AB(4), the position of the E→Z switched AB subunits within the oligomer strongly influences the observed changes in the absorption spectra (Figure 6). For example, if the (*Z*)‐AB subunits are located at the end of the chain as in (*ZEEE*)‐ or (*ZZEE*)‐*para*‐AB(4), the resulting absorption spectrum is dominated by the spectroscopic signature of the remaining intact all‐(*E*) *para*‐AB chain. For instance, (*ZEEE*)‐*para*‐AB(4) has an intact all‐(*E*) chain of three AB subunits and possesses thus an absorption spectrum similar to all‐(*E*) *para*‐AB(3). Similarly, (*ZZEE*)‐ *para*‐AB(4) has an intact all‐(*E*) chain of only two AB subunits, and its corresponding absorption spectrum resembles thus the one of the all‐(*E*) *para*‐AB(2) oligomer. This can easily be seen by comparing the absorption spectra in Figures 2 and 6, where the absorption maxima are located at 3.4 and 3.7 eV for all‐(*E*) *para*‐AB(3) and all‐(*E*) *para*‐AB(2) and at 3.4 and 3.6 eV for (*ZEEE*)‐*para*‐AB(4) and *ZZEE*‐*para*‐AB(4), respectively.

However, in cases for which the E→Z switched AB subunits are located in the middle of the oligomer, the situation becomes slightly more complicated, since it leads to two separated all‐(*E*) chains, each of which generates a peak in the absorption spectrum corresponding to its length. In (*EZEE*)‐*para*‐AB(4), for example, this results in two separate absorption bands corresponding to the one of (*E*)‐AB and the all‐(*E*) *para*‐AB(2) absorption (see red curve in Figure 6). They originate from the two independent π→π* transitions localized on the (*E*)‐AB(1) and the all‐(*E*) *para*‐AB(2) chain present owing to the breaking of the *π*‐conjugation by E→Z switching of the second AB subunit in the all‐(*E*) *para*‐AB(4) oligomer. Therefore, the peak with the largest oscillator strength in the absorption spectrum of E→Z switched *para*‐AB(*n*) oligomers originates from the π→π* excitation of the longest remaining all‐(*E*) *para*‐AB(*n*) chain. Based on these findings, it is possible to read off the isomerization state from the absorption spectrum of the respective oligomer. Moreover, the strong dependence of the absorption spectrum on the isomerization state of the individual AB subunits also demonstrates once more the strong coupling of the AB subunits in the *para*‐connected AB(*n*) oligomers.

**Figure 7:**
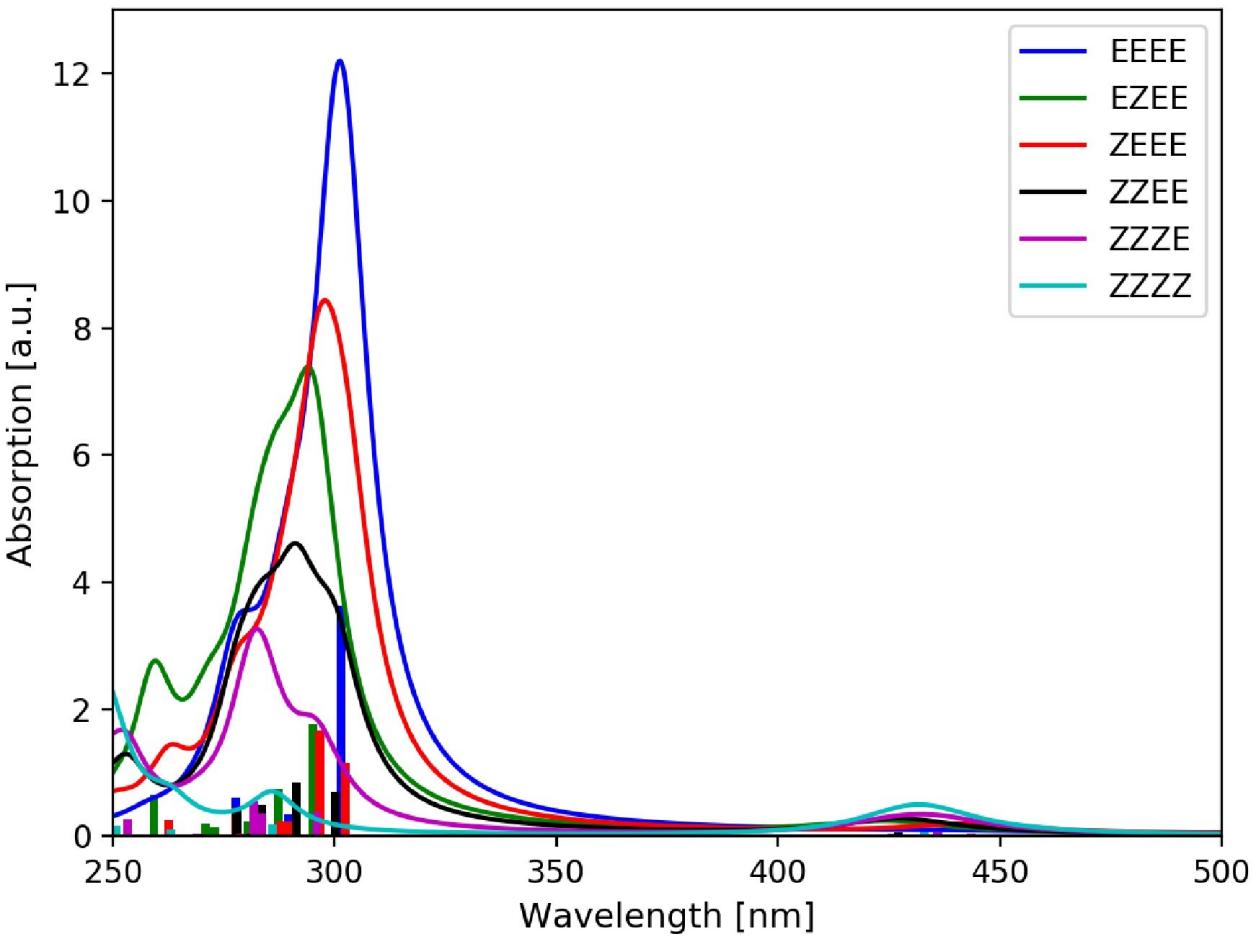
Simulated absorption spectra (TDDFT/CAM‐B3LYP) of *meta*‐AB(4)s with varying numbers of (*Z*)‐AB units. All (*Z*)‐isomers considered here are of *M* chirality.The stick spectra were convoluted with a Lorentzian function of 0.2 eV FWHM.

For the *meta*‐connected AB(*n*) oligomers, the absorption spectra of the E→Z switched isomers yield a different and much simpler picture. This owes to the fact that the absorption spectra of the all‐(*E*) *meta*‐AB(*n*) oligomers resemble *n*‐tuples of (*E*)‐AB, and that the main absorption peak originating from the bright π→π* excitation hardly changes in energy with increasing *n*, and that the all‐(*E*) *meta*‐AB(*n*) can be seen as *n* practically independent azobenzenes. Therefore, by E→Z switching of a single AB subunit, the oscillator strength of the main peak is reduced by approximately one equivalent of the absorption strength of a single (*E*)‐AB unit and a (*Z*)‐AB spectrum is added. As a consequence of the independence of the AB subunits in, for example, all‐(*E*) *meta*‐AB(4), the absorption spectrum of a singly E→Z switched *meta*‐AB(4) is quasi independent of the position of the isomerization. This becomes apparent when comparing the absorption spectra of the (*EZEE*)‐ and (*ZEEE*)‐ *meta*‐AB(*n*) isomers, displayed in green and red in Figure 7, respectively. Accordingly, the absorption spectrum of a doubly E→Z switched *meta*‐AB(4) looks very much like the sum of two isolated (*E*)‐AB and two isolated (*Z*)‐AB spectra. For the *meta*‐connected AB(*n*) oligomers, it is thus very difficult if not impossible to read off the specific isomerization state of an oligomer from the absorption spectrum. It may be possible to identify the number of isomerized AB units by proper intensity calibration of the spectra, but it is generally not possible to locate the position of the isomerization as in the *para*‐connected ones.

Since the AB subunits keep their individuality in the *meta*‐connected oligomer units and their individual spectroscopic properties, the oligomers will be equally well switchable as individual azobenzenes, and one can expect to reach similar photostationary equilibria. Consequently, *meta*‐*oligo*‐azobenzenes are expected to be ideal candidates to achieve high switching ratios, necessary for efficient applications.

## Conclusions

The electronic structure and the spectroscopic properties of linear *meta*‐ and *para*‐connected *oligo*‐azobenzenes AB(*n*)s with up to four AB subunits have been theoretically investigated at the level of density functional theory. The absorption spectra, detachment and attachment densities as well as exciton properties of the all‐(*E*) isomers have been studied in detail and spectroscopic signatures of possible E→Z switching processes have been identified. Our computations strongly suggest, that the individual switching ability of the individual AB subunits is conserved within the *meta*‐connected AB(*n*) oligomers. In the *para*‐connected oligomers, the AB subunits loose their individuality and are most likely no longer photoswitchable, or at least with strongly decreasing quantum yield with increasing number of AB subunits. The loss of individuality coincides with a drastic increase of the exciton size and lowering of the vertical excitation energy in the *para*‐AB(*n*). In other words, the more delocalized the initial excitation is, the less efficient appears a switching process to be, since prior to switching localization and concentration of the excitation at a specific switching site needs to occur. Hence, the exciton size may be a valid and easily computable parameter to judge the individuality of connected individual photoswitches. Our findings further corroborate the success of the so‐called “ *meta*‐rule”, which states that *meta*‐connected AB subunits keep their individual functionality.[^18^, ^19^, ^20^, ^21^]

We hope to encourage the resumption of further synthetic and spectroscopic efforts on these fascinating linear azobenzene‐based oligomers, since we expect the *meta*‐AB(*n*) oligomers and polymers to be of great use as functional building blocks in photoswitchable devices. Moreover, by chemical modification of selected benzene rings in the *meta*‐AB(*n*) by, for example, substitution with fluorine atoms or other suitable ligands, it should be possible to shift the absorption spectrum of isolated AB subunits such that they become individually addressable and switchable following the strategy previously applied to star‐like trisazobenzenes.[^22^, ^23^] Thereby, stepwise switching of different AB subunits within one polymer may become feasible opening a route to fascinating photochemistry.

## Computational Details

For the evaluation of ground state as well as excited state properties of the AB(*n*) systems Kohn‐Sham DFT and its linear‐response time‐dependent variant TDDFT at the CAM‐B3LYP/6‐311G*+D3(BJ) level of theory were employed as implemented within the Q‐Chem 5.2 program package.^[33]^ Previous studies[^24^, ^25^] showed this methodology to be sufficiently accurate, giving qualitatively correct results: it reproduces consistently blue‐shifted absorption spectra by approx. 0.3 eV, however, with the correct excited‐state ordering and relative energies, as well as correct shapes of the corresponding potential energy surfaces. Ground state geometry optimizations were done for all relevant isomers (according to the calculated Boltzmann populations), which were confirmed to be minima with only real harmonic frequencies. The absorption spectra were generated from the first energetically lowest 20 singlet excited states. The Libwfa library^[32]^ was employed for the generation of detachment and attachment densities of the low‐lying relevant states. The detachment density describes that part of the ground state density that is removed upon the excitation and re‐added as the attachment density. For the evaluation of the electronic structure of the excited states within the appealing exciton picture the corresponding analysis tools implemented in Q‐Chem 5.2 were employed.[^34^, ^35^]

## Conflict of Interests

The authors declare no conflict of interest.

1

## Supporting information

As a service to our authors and readers, this journal provides supporting information supplied by the authors. Such materials are peer reviewed and may be re‐organized for online delivery, but are not copy‐edited or typeset. Technical support issues arising from supporting information (other than missing files) should be addressed to the authors.

Supporting Information

## Data Availability

The data that support the findings of this study are available in the supplementary material of this article.
