# Supplementary material for: Meta‐Connected Oligo‐Azobenzenes Outperform Their Para Counterparts
Source: Chemphyschem. 2024 Nov 29;26(4):e202400799. doi: 10.1002/cphc.202400799 (PMC11832058; doi:10.1002/cphc.202400799)
Supplement: Supplementary file 1 — Supporting Information [file CPHC-26-e202400799-s001.pdf]

ChemPhysChem

Supporting Information

Meta-Connected *Oligo*-Azobenzenes Outperform Their *Para* Counterparts

Nils Oberhof, Leon Kambiz Paschai Darian, and Andreas Dreuw*

Meta-Connected *Oligo*-Azobenzenes Outperform Their *Para* Counterparts

Nils Oberhof, Leon Kambiz Paschai Darian, Andreas Dreuw

Supporting Information

Additionally to the (*E/Z*)-isomers already given in the main text, the (EZZE)- and (ZEEZ)-conformers of the *meta*-AB(4) were calculated. Their simulated absorption spectra have been omitted in the main text for visual and contextual clarity.

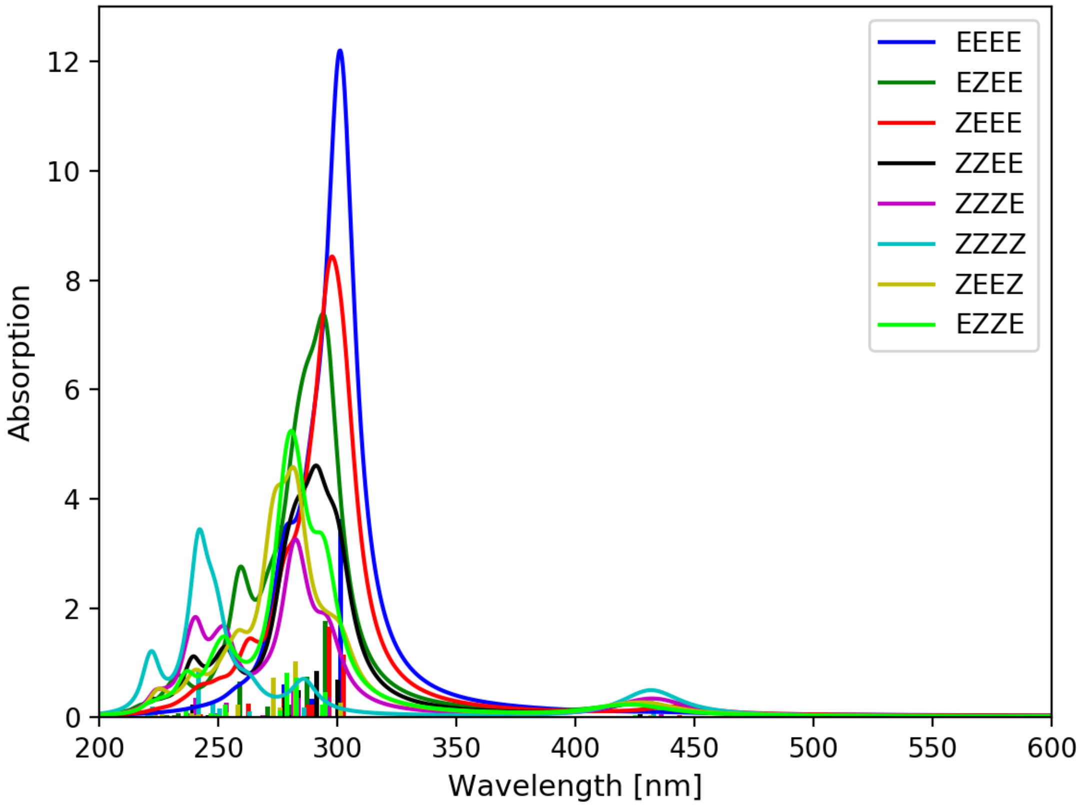

Figure 1: Simulated absorption spectra of the all-(E) meta-AB(n)s convoluted with a Lorentzian function with 0.2 eV FWHM.

Ground-state geometries of relevant species:

AB(1)
24
E = -572.582946774248

H	2.1919319006	-2.2595211256	-0.0000382471
C	2.6242355748	-1.2660345415	-0.0000210765
C	1.7656194042	-0.1742197528	0.0000049589
C	2.2796604453	1.1213019607	0.0000301166
H	1.5916339887	1.9559024449	0.0000512988
C	3.6488905906	1.3097423859	0.0000275639
H	4.0531700231	2.3157211044	0.0000476792
C	4.5099506649	0.2157435816	0.0000000529
H	5.5828369117	0.3715200675	-0.0000012747
C	3.9966451763	-1.0724998547	-0.0000245460
H	4.6636976284	-1.9268594608	-0.0000455506
N	0.3806510530	-0.4849582698	0.0000078289
N	-0.3792377994	0.4953876071	0.0000085388
C	-1.7637317925	0.1820727552	0.0000050666
C	-2.2743396473	-1.1154314368	0.0000289634
H	-1.5845290188	-1.9483848490	0.0000500577
C	-3.6426887235	-1.3116497432	0.0000252531
H	-4.0413548571	-2.3200398406	0.0000445444
C	-4.5098315012	-0.2229270515	-0.0000021836
H	-5.5820378157	-0.3843099402	-0.0000044730
C	-4.0020298882	1.0674072040	-0.0000253755
C	-2.6298535827	1.2688160039	-0.0000207262
H	-2.2050595812	2.2654445406	-0.0000370336
H	-4.6742307825	1.9177751713	-0.0000462729

meta-AB(2)
36
E = -913.002415801551

H	5.7673444623	-1.6214402347	0.3216718927
C	5.8019515948	-0.5409621455	0.2494948853
C	4.6025670607	0.1505372412	0.1371976654
C	4.6004230315	1.5410974460	0.0409884424
H	3.6539849388	2.0574224375	-0.0465476946
C	5.8009536030	2.2253797100	0.0590300545
H	5.8049477019	3.3068713153	-0.0160274727
C	7.0034191294	1.5330927916	0.1723836285
H	7.9410695886	2.0770839611	0.1856609060
C	7.0034966494	0.1495839877	0.2679633648
H	7.9380863135	-0.3921108636	0.3566252205
N	3.4311087446	-0.6506353550	0.1281235490
N	2.3638070076	-0.0240637912	0.0479051976
C	1.1967967992	-0.8327519401	0.0375212373
C	1.2082361221	-2.2252275383	0.0711089925

H	2.1588470631	-2.7393526898	0.1072427529
C	0.0098956433	-2.9213847219	0.0571654790
H	0.0186711497	-4.0052011575	0.0809850781
C	-1.1995086016	-2.2470359100	0.0129025610
H	-2.1424283246	-2.7765797598	0.0026894590
C	-1.2096937896	-0.8547507107	-0.0202118527
C	-0.0127681344	-0.1521786979	-0.0114965610
H	-0.0239270380	0.9298527501	-0.0400147032
N	-2.3918338967	-0.0683627781	-0.0662776281
N	-3.4481895874	-0.7173175288	-0.0331729728
C	-4.6364797572	0.0559065938	-0.0813192821
C	-4.6663527931	1.4462767730	-0.1795341221
H	-3.7311880946	1.9879075895	-0.2220782975
C	-5.8837338376	2.1001896595	-0.2210559788
H	-5.9114160413	3.1812856614	-0.2980174231
C	-7.0728234170	1.3785632719	-0.1652713634
C	-7.0423783477	-0.0044849533	-0.0673937813
C	-5.8239043802	-0.6647708561	-0.0261769700
H	-5.7676847276	-1.7441458425	0.0492881200
H	-7.9667464282	-0.5689465787	-0.0234721967
H	-8.0234568175	1.8989794732	-0.1988923114

meta-AB(3)
48
E = -1253.42177503148

H	8.5726523855	-1.9063104273	0.1937784725
C	8.6842791721	-0.8430240460	0.0181569821
C	7.5360866895	-0.0686961298	-0.0879621445
C	7.6325358576	1.3030165637	-0.3161656818
H	6.7239982676	1.8843344300	-0.3972129142
C	8.8798225008	1.8857352376	-0.4358055162
H	8.9608636615	2.9518087467	-0.6152304121
C	10.0311975637	1.1105251469	-0.3288003445
H	11.0060542570	1.5750510481	-0.4250322164
C	9.9331702801	-0.2539441506	-0.1011520338
H	10.8280531977	-0.8596897234	-0.0177086759
N	6.3086632085	-0.7693554246	0.0426650603
N	5.2904762107	-0.0640381404	-0.0192540319
C	4.0653056551	-0.7709844432	0.1018157511
C	3.9662141424	-2.1552610484	0.2211017056
H	4.8724529728	-2.7452589934	0.2281966174
C	2.7174722264	-2.7472261689	0.3289293423
H	2.6406734235	-3.8245023042	0.4230204287
C	1.5659351910	-1.9767778738	0.3183184496
H	0.5848025721	-2.4243942251	0.4009132650
C	1.6669777522	-0.5928333199	0.1986821187
C	2.9145290879	0.0058321845	0.0926340342
H	2.9892314166	1.0818182161	0.0012877554

N	0.5543552277	0.2890055995	0.1856905310
N	-0.5533382451	-0.2682038295	0.1832289350
C	-1.6673246992	0.6111883036	0.1899803493
C	-1.5741745197	1.9960407181	0.3063476407
H	-0.5957799166	2.4487836052	0.3910090145
C	-2.7297379100	2.7615604474	0.3121666750
H	-2.6586027897	3.8393008066	0.4040766814
C	-3.9751402347	2.1636894395	0.2029625245
H	-4.8854215574	2.7476608814	0.2070781869
C	-4.0659296464	0.7787167546	0.0870378155
C	-2.9128724224	0.0068380402	0.0821971055
H	-2.9864807102	-1.0697305284	-0.0059495862
N	-5.2885311070	0.0654630121	-0.0329598464
N	-6.3091037089	0.7669424196	0.0339410116
C	-7.5348482333	0.0642740396	-0.0942658753
C	-7.6318602413	-1.3051863033	-0.3370390595
H	-6.7241373275	-1.8851111049	-0.4343011853
C	-8.8790561651	-1.8907111312	-0.4492498539
H	-8.9587689590	-2.9551028266	-0.6396191732
C	-10.0320206665	-1.1217922089	-0.3200801179
H	-11.0064070541	-1.5889702946	-0.4097971026
C	-9.9350339140	0.2403603354	-0.0787572504
C	-8.6861768546	0.8326272461	0.0321747914
H	-8.5782451746	1.8943834488	0.2185655175
H	-10.8313689281	0.8415285752	0.0220034105

meta-AB(4) EEEE
60
E = -1593.84116421300

H	11.5763309057	-1.5410963222	0.2800034096
C	11.6312567341	-0.4753224825	0.0931049676
C	10.4434965856	0.2344415400	-0.0280928086
C	10.4672131213	1.6070501599	-0.2701221268
H	9.5292207310	2.1377747979	-0.3622823924
C	11.6818325190	2.2552203793	-0.3886134998
H	11.7061094302	3.3223385373	-0.5781045234
C	12.8727322146	1.5445669572	-0.2673585273
H	13.8216251475	2.0601616965	-0.3630621219
C	12.8471181445	0.1791184943	-0.0260601703
H	13.7728620641	-0.3765190329	0.0687580922
N	9.2553052914	-0.5301635002	0.1052668135
N	8.1998437708	0.1146767591	0.0138473191
C	7.0153745390	-0.6577161848	0.1414648767
C	6.9949942299	-2.0393171293	0.3179326638
H	7.9338343751	-2.5737619672	0.3664992100
C	5.7812635525	-2.6993410256	0.4290130164
H	5.7658035655	-3.7744125982	0.5676785352
C	4.5872409385	-1.9996919612	0.3656648775
H	3.6327409196	-2.5010815941	0.4512385592

C	4.6093041664	-0.6183112908	0.1888074949
C	5.8214462321	0.0485135461	0.0788909528
H	5.8348144307	1.1223251523	-0.0568839337
N	3.4462557192	0.1932533023	0.1175898957
N	2.3748642859	-0.4303680893	0.1427477882
C	1.2084491547	0.3758631291	0.0871701335
C	1.2119267019	1.7687163048	0.0977532512
H	2.1588666399	2.2888333704	0.1459116858
C	0.0095234596	2.4570088551	0.0470026642
H	0.0115497868	3.5408944656	0.0554211119
C	-1.1952468121	1.7746564677	-0.0143226751
H	-2.1409259175	2.2980746071	-0.0546690289
C	-1.1957543848	0.3819896250	-0.0243512181
C	0.0043995057	-0.3126089673	0.0268184941
H	-0.0000062017	-1.3951846168	0.0192414592
N	-2.3661169230	-0.4180611827	-0.0917809903
N	-3.4351511474	0.2099791090	-0.0683557708
C	-4.6009281023	-0.5961662666	-0.1513709683
C	-4.5829552518	-1.9777032178	-0.3287815473
H	-3.6299214676	-2.4827836690	-0.4051982557
C	-5.7782124101	-2.6744382049	-0.4045542020
H	-5.7649618263	-3.7494929476	-0.5433258960
C	-6.9902873382	-2.0110359604	-0.3059572293
H	-7.9311739464	-2.5409681523	-0.3647398653
C	-7.0071779628	-0.6295942054	-0.1294971959
C	-5.8133536710	0.0741820823	-0.0533339019
H	-5.8294042166	1.1479943634	0.0827007763
N	-8.1951157013	0.1420358589	-0.0165382526
N	-9.2451391293	-0.5082637475	-0.1291229065
C	-10.4423592120	0.2428290094	-0.0138815650
C	-11.6191549891	-0.4802621174	-0.1683176658
H	-11.5472619988	-1.5435518999	-0.3634008435
C	-12.8466856671	0.1568536856	-0.0724987928
H	-13.7627828775	-0.4097083763	-0.1940951778
C	-12.8967610150	1.5195894261	0.1799593092
H	-13.8543533863	2.0223047580	0.2573890667
C	-11.7182745625	2.2436322669	0.3357783621
H	-11.7614694434	3.3086269930	0.5340350401
C	-10.4919553769	1.6125894017	0.2400328906
H	-9.5646716189	2.1566032678	0.3584385153

meta-AB(4) ZEEE
60
E = -1593.81852934809

H	11.1900878664	-2.0363042027	-0.3033220453
C	11.3181532704	-0.9712507758	-0.1512028369
C	10.1825103275	-0.1847316529	-0.0070598641
C	10.3006891022	1.1903051368	0.1888247328
H	9.4014935344	1.7816439309	0.2969903901

C	11.5568235617	1.7639030038	0.2399693465
H	11.6544914880	2.8328976772	0.3915909748
C	12.6957469843	0.9760413597	0.0975649874
H	13.6778338968	1.4333970699	0.1390401155
C	12.5760776219	-0.3916265519	-0.0981039926
H	13.4612564921	-1.0070262912	-0.2098436292
N	8.9439510805	-0.8749640008	-0.0755456118
N	7.9378240800	-0.1645334279	0.0690799592
C	6.7002281480	-0.8560274051	-0.0033932248
C	6.5757726376	-2.2284664034	-0.2064770647
H	7.4720417381	-2.8239137917	-0.3149902591
C	5.3150043261	-2.8022508565	-0.2635440770
H	5.2186787803	-3.8709079366	-0.4183475510
C	4.1762939280	-2.0250284445	-0.1241397232
H	3.1862494473	-2.4585133507	-0.1678111120
C	4.3031469759	-0.6530023198	0.0785367438
C	5.5627081059	-0.0739589415	0.1433483056
H	5.6570245108	0.9921883627	0.3047137680
N	3.2085758818	0.2361031745	0.2364805591
N	2.0891117890	-0.2748608789	0.0836986465
C	0.9995092029	0.6201020315	0.2511292011
C	1.1302016997	1.9403863372	0.6748156215
H	2.1178419717	2.3227007055	0.8931521927
C	0.0014716368	2.7340315492	0.8058100814
H	0.1033298591	3.7615834572	1.1352718343
C	-1.2552525186	2.2283389787	0.5159361991
H	-2.1447833199	2.8365839040	0.6068246555
C	-1.3838963201	0.9072269613	0.0931278211
C	-0.2583013761	0.1054032600	-0.0339584638
H	-0.3622245109	-0.9208651786	-0.3628763025
N	-2.6209634593	0.2989172832	-0.2495178775
N	-3.6138544979	1.0279313893	-0.1075634844
C	-4.8505012384	0.4438323292	-0.4854130625
C	-4.9883354861	-0.8390963121	-1.0132282859
H	-4.1088037261	-1.4575084361	-1.1279768104
C	-6.2391653763	-1.2794008378	-1.4068722160
H	-6.3503065630	-2.2664024257	-1.8408186477
C	-7.3595221615	-0.4727896724	-1.2555407441
H	-8.3296628557	-0.8278964274	-1.5770002616
C	-7.2222191189	0.7967841782	-0.7039574126
C	-5.9626081851	1.2616739434	-0.3494969750
H	-5.8441784524	2.2728318205	0.0187892828
N	-8.2791047305	1.7642483544	-0.6321293345
N	-9.4277009779	1.5053124766	-0.2610467855
C	-9.8213370396	0.2375701206	0.2754259811
C	-10.9755729116	-0.3292169394	-0.2514204099
H	-11.4794100490	0.1738262497	-1.0686784697
C	-11.4587677013	-1.5189636260	0.2682955463
H	-12.3472165834	-1.9708062518	-0.1581295959

C	-10.8223067105	-2.1161223522	1.3479928928
H	-11.2116490784	-3.0364271869	1.7678284655
C	-9.6989721022	-1.5185034885	1.9037065670
H	-9.2113758635	-1.9704844661	2.7600145374
C	-9.1895471671	-0.3467704051	1.3674877167
H	-8.3097566757	0.1169741025	1.7966121741

meta-AB(4) ZZEE
60
E = -1593.79681457475

H	-9.7603492030	1.2985427985	1.8772520674
C	-9.6392317914	1.8387191404	0.9458727770
C	-8.4869751986	1.6194605193	0.2011254579
C	-8.2933150291	2.2920632192	-1.0044734364
H	-7.3904168761	2.1043015336	-1.5697927810
C	-9.2576989020	3.1741386259	-1.4541420395
H	-9.1129860903	3.6965678570	-2.3931510452
C	-10.4146957380	3.3907575199	-0.7110626056
H	-11.1686709490	4.0807636847	-1.0731839441
C	-10.6045377471	2.7232142193	0.4895952551
H	-11.5055659096	2.8885673393	1.0691148484
N	-7.5774854442	0.6767287072	0.7436600154
N	-6.5524177592	0.4873426868	0.0721423766
C	-5.6571666370	-0.4726043919	0.6112391600
C	-5.9211600650	-1.2474507098	1.7383114546
H	-6.8656090543	-1.1205857326	2.2500206902
C	-4.9791692641	-2.1658856120	2.1742979569
H	-5.1879844815	-2.7753764525	3.0461322854
C	-3.7738630665	-2.3145165429	1.5063363952
H	-3.0273148939	-3.0234665279	1.8381059074
C	-3.5139003378	-1.5373955949	0.3801563865
C	-4.4606808634	-0.6295826388	-0.0719975530
H	-4.2622254209	-0.0358966995	-0.9552542223
N	-2.3145633903	-1.5973589494	-0.3733329847
N	-1.4157148240	-2.2873166078	0.1296403252
C	-0.2134068207	-2.3299276356	-0.6224331944
C	-0.0938360278	-1.8685061803	-1.9320227493
H	-0.9652014760	-1.4626318401	-2.4273448309
C	1.1312435988	-1.9490348929	-2.5682700303
H	1.2298054206	-1.6114150250	-3.5934549640
C	2.2451669750	-2.4428395350	-1.9015407411
H	3.2083392954	-2.4826808621	-2.3950844372
C	2.1163434450	-2.8875606293	-0.5923719178
C	0.8781784211	-2.8800508165	0.0303979171
H	0.7614750830	-3.2684773939	1.0345584873
N	3.2098679525	-3.4676981047	0.1277603110
N	4.2607464075	-2.8493491746	0.3228097936
C	4.4271377419	-1.4724939580	-0.0495786157
C	3.5441760954	-0.4695564804	0.3388062174

H	2.6392620367	-0.7175965858	0.8772609185
C	3.8294607263	0.8471622102	0.0160252791
H	3.1370963013	1.6300105889	0.3026745869
C	4.9914568462	1.1745605440	-0.6637512158
H	5.2150673743	2.2051694433	-0.9076552093
C	5.8856598138	0.1674739248	-1.0094275525
C	5.5994339155	-1.1560922067	-0.7178109795
H	6.2929835432	-1.9403506338	-0.9969006396
N	7.0740205857	0.4315755056	-1.7663782674
N	7.9315303523	1.2313206391	-1.3778990161
C	7.8644767455	1.8622582376	-0.0912688757
C	7.7274616462	1.1451830748	1.0926822338
H	7.6025437724	0.0702629028	1.0666829794
C	7.7571632735	1.8174363216	2.3030935267
H	7.6543628268	1.2590977873	3.2264896837
C	7.9079902595	3.1974484431	2.3356340830
H	7.9202238668	3.7193449242	3.2854431508
C	8.0644134428	3.9045733421	1.1512190047
H	8.2006586929	4.9796957959	1.1730260909
C	8.0698609238	3.2349043191	-0.0616481975
H	8.2247777737	3.7607100738	-0.9966570252

meta-AB(4) EZEE
60
E = -1593.81869019084

H	11.0552498338	-0.1498317863	-0.9672887114
C	10.9065436411	0.8070719761	-0.4813905406
C	9.6189363767	1.1570824633	-0.0952974913
C	9.3817046444	2.3785714864	0.5331079192
H	8.3704346061	2.6289201630	0.8240865560
C	10.4378564810	3.2378512740	0.7688313772
H	10.2591371550	4.1889420776	1.2575492649
C	11.7287108801	2.8875661724	0.3829099322
H	12.5521546674	3.5668312870	0.5722869572
C	11.9627724277	1.6721054027	-0.2425681730
H	12.9669704926	1.3971139522	-0.5436873877
N	8.6105178151	0.2013992023	-0.3839353824
N	7.4649020874	0.5196978029	-0.0311511015
C	6.4627852903	-0.4430887769	-0.3223754502
C	6.7010941602	-1.6484320166	-0.9786310967
H	7.7088161756	-1.8803378551	-1.2949909658
C	5.6506882953	-2.5217465816	-1.2127764378
H	5.8353355841	-3.4583523714	-1.7265201255
C	4.3665675209	-2.2105061435	-0.7968396314
H	3.5377709369	-2.8834851992	-0.9695950704
C	4.1301571334	-1.0053797034	-0.1397174992
C	5.1759693441	-0.1223616245	0.0895010615
H	4.9882647733	0.8155726629	0.5964308780

N	2.8536703880	-0.5929716653	0.3273518080
N	1.9581505852	-1.4411066221	0.2006700741
C	0.6769244854	-1.0407080372	0.6610811594
C	0.3696887437	0.2310603116	1.1427115720
H	1.1463336718	0.9828120675	1.1736403012
C	-0.9133162627	0.4957361511	1.5884321405
H	-1.1555547495	1.4770902659	1.9795548342
C	-1.9011487062	-0.4796492230	1.5366624782
H	-2.9031569491	-0.2635397429	1.8857737015
C	-1.5930719738	-1.7395759218	1.0372438419
C	-0.2986089605	-2.0262563645	0.6278566720
H	-0.0439137396	-3.0219129564	0.2863550233
N	-2.5080326110	-2.8435227037	1.0649335630
N	-3.6588847712	-2.7894620540	0.6219895989
C	-4.1804946204	-1.6634242502	-0.0944214061
C	-3.5230724907	-1.0550396860	-1.1580950403
H	-2.5266676311	-1.3732504098	-1.4359598140
C	-4.1612610693	-0.0490117180	-1.8712570585
H	-3.6515234866	0.4174978684	-2.7063965137
C	-5.4369136124	0.3622132336	-1.5288032600
H	-5.9443664862	1.1468138258	-2.0731827461
C	-6.1003746299	-0.2690630322	-0.4784195534
C	-5.4835632907	-1.2987549832	0.2155516038
H	-6.0238878045	-1.8188777034	0.9967504355
N	-7.4305028917	0.0291132135	-0.0809247825
N	-7.9135075948	1.0461379847	-0.6008175320
C	-9.2555808983	1.3260595238	-0.2367751616
C	-9.7476901345	2.5593248994	-0.6459394762
H	-9.0900258604	3.2185032018	-1.1997039269
C	-11.0505451746	2.9229961182	-0.3417562369
H	-11.4314811562	3.8877891718	-0.6563094737
C	-11.8652809290	2.0465910350	0.3596917043
H	-12.8869319882	2.3250685971	0.5928481178
C	-11.3759480391	0.8061299372	0.7583021408
H	-12.0178464922	0.1199476287	1.2992107755
C	-10.0749890525	0.4422028783	0.4648212909
H	-9.6755394119	-0.5176854612	0.7635799741

meta-AB(4) ZZZE
60
E = -1593.77444041277

H	-8.2630217840	-1.5767659690	2.3199754929
C	-8.3521480210	-2.0815338562	1.3655328126
C	-7.5719738536	-1.6346914032	0.3061254963
C	-7.6556247393	-2.2598572378	-0.9376715475
H	-7.0383575484	-1.8971719048	-1.7482198354
C	-8.5188099579	-3.3252718182	-1.1089926856
H	-8.5851106138	-3.8145305377	-2.0742909776
C	-9.3020431160	-3.7725237182	-0.0487085593

H	-9.9765987477	-4.6094972672	-0.1904355491
C	-9.2185122602	-3.1500443107	1.1878119636
H	-9.8274557342	-3.4974956175	2.0143066862
N	-6.7128328681	-0.5423738285	0.5891076677
N	-6.0405703351	-0.1439264476	-0.3735840633
C	-5.1644362162	0.9350211592	-0.0875298343
C	-5.0930922939	1.5906267562	1.1405488854
H	-5.7538750153	1.2811511272	1.9384271719
C	-4.1891834324	2.6243021333	1.3046245101
H	-4.1388370243	3.1506604125	2.2505989956
C	-3.3295949851	2.9868781819	0.2747416594
H	-2.6079484820	3.7826719336	0.4127886388
C	-3.3994670553	2.3166155427	-0.9392077445
C	-4.3456324469	1.3231706475	-1.1365835869
H	-4.4325977250	0.8254612280	-2.0947610526
N	-2.5901156958	2.6846833100	-2.0630995852
N	-1.3563271196	2.6588512777	-2.0195477645
C	-0.6288487039	2.1499451103	-0.8912077506
C	-0.8979996798	0.9199170630	-0.2978746616
H	-1.7373765128	0.3243343180	-0.6295266223
C	-0.0857286392	0.4691407054	0.7294861409
H	-0.3030711568	-0.4789180132	1.2071368116
C	1.0070539611	1.2109354357	1.1461808214
H	1.6421553484	0.8496215007	1.9442677080
C	1.3008266752	2.4100174910	0.5064735783
C	0.4741221408	2.8919964436	-0.4952435484
H	0.6974759319	3.8311987368	-0.9868182687
N	2.4104876255	3.2301953637	0.8996881541
N	3.5628423966	2.7842131930	0.9276687951
C	3.8661592880	1.4613190281	0.4596631982
C	3.5704574809	1.0547130195	-0.8368242919
H	3.0590996645	1.7256967119	-1.5147303577
C	3.9092653578	-0.2264984494	-1.2391214070
H	3.6620095796	-0.5560296140	-2.2415327264
C	4.5520594577	-1.0947195821	-0.3723821915
H	4.8017296081	-2.0983951029	-0.6902708361
C	4.8845215794	-0.6622698510	0.9075034697
C	4.5368839587	0.6138039787	1.3267212477
H	4.7684376573	0.9344662484	2.3354033866
N	5.4423800475	-1.5340781318	1.9008759900
N	6.4280024713	-2.2486002799	1.6926327545
C	7.1993053090	-2.1730315644	0.4878879000
C	7.7411878426	-0.9801727157	0.0219733097
H	7.5303996633	-0.0512179010	0.5368741422
C	8.5525445646	-0.9931414711	-1.1004693906
H	8.9791390655	-0.0647715957	-1.4623726610
C	8.8118424766	-2.1843955352	-1.7653427185
H	9.4395216292	-2.1868575775	-2.6488826518
C	8.2836925261	-3.3747362319	-1.2837097451

H	8.4983647635	-4.3094737738	-1.7889737240
C	7.4988573692	-3.3744473467	-0.1418735866
H	7.1038266780	-4.2952758105	0.2713659568

para-AB(2)
36
E = -913.003017080219

H	4.7211529097	-1.9537876077	0.0000595631
C	5.4114288515	-1.1211425937	0.0000276696
C	6.7795590596	-1.3154978538	0.0000266277
H	7.1795572115	-2.3232977950	0.0000586082
C	7.6444380830	-0.2248093333	-0.0000153723
H	8.7166696136	-0.3852726016	-0.0000157908
C	7.1354148355	1.0653267723	-0.0000561694
H	7.8068676562	1.9160600517	-0.0000889071
C	5.7633853635	1.2653374100	-0.0000547783
H	5.3350076646	2.2603973878	-0.0000854158
C	4.9006763228	0.1761062284	-0.0000134289
N	3.5167566216	0.4873686313	-0.0000154214
N	2.7565714153	-0.4937553520	0.0000138986
C	1.3739546869	-0.1818314302	0.0000112370
C	0.5107737344	-1.2761114481	0.0000182696
H	0.9425462369	-2.2696952468	0.0000245629
C	-0.8570016791	-1.0911960581	0.0000176851
H	-1.5415613263	-1.9282210940	0.0000221039
C	-1.3722260191	0.2031204946	0.0000121414
C	-0.5106221194	1.2977934539	0.0000084639
H	-0.9444480938	2.2904675750	0.0000072356
C	0.8573729463	1.1124159656	0.0000065680
H	1.5407056068	1.9501284827	0.0000025136
N	-2.7565265677	0.5111879018	0.0000152819
N	-3.5115993124	-0.4738622703	-0.0000118018
C	-4.8971151688	-0.1727106498	-0.0000116871
C	-5.7503823669	-1.2698577806	-0.0000599134
H	-5.3137590068	-2.2613580298	-0.0000945503
C	-7.1244565685	-1.0843105359	-0.0000626805
H	-7.7870986671	-1.9420853824	-0.0001009446
C	-7.6466233843	0.2003772012	-0.0000157678
C	-6.7924751659	1.2995465997	0.0000335647
H	-7.2031651778	2.3029890749	0.0000707689
C	-5.4219763844	1.1193437705	0.0000357112
H	-4.7413059878	1.9596975737	0.0000736259
H	-8.7206059928	0.3494826383	-0.0000167376

para-AB(3)
48
E = -1253.42302054393

H	7.8545878985	-1.9630312880	-0.0039373493
C	8.5452147129	-1.1306782235	-0.0041640274

C	9.9132535838	-1.3256541972	-0.0065599473
H	10.3129037007	-2.3335670201	-0.0082889913
C	10.7785954307	-0.2353015456	-0.0067641365
H	11.8507261296	-0.3963383454	-0.0086445120
C	10.2703763266	1.0551676553	-0.0045521126
H	10.9423076022	1.9054940676	-0.0046984720
C	8.8984962805	1.2558830069	-0.0021489221
H	8.4705550227	2.2511150116	-0.0003695425
C	8.0353054782	0.1669594135	-0.0019385118
N	6.6518748952	0.4791742550	0.0007152160
N	5.8901222710	-0.5008003635	0.0009015880
C	4.5079628250	-0.1860018650	0.0022186730
C	3.6423979900	-1.2785801141	0.0023841016
H	4.0720950284	-2.2730355738	0.0014753001
C	2.2751827435	-1.0906409539	0.0036961952
H	1.5889110776	-1.9262456370	0.0038853118
C	1.7630788084	0.2053278892	0.0048650161
C	2.6271774531	1.2982974313	0.0047167337
H	2.1955516521	2.2918939091	0.0056885264
C	3.9945887300	1.1095788058	0.0034119034
H	4.6800046577	1.9455200963	0.0033050649
N	0.3808980496	0.5165619193	0.0064292851
N	-0.3779275534	-0.4666714718	0.0064360097
C	-1.7601777516	-0.1596429280	0.0049427255
C	-2.6190974699	-1.2575570484	0.0048510858
H	-2.1833850024	-2.2493736414	0.0058083100
C	-3.9873934072	-1.0778092507	0.0036253081
H	-4.6688582945	-1.9172788081	0.0035698359
C	-4.5074221048	0.2146519844	0.0024555384
C	-3.6502489604	1.3129736660	0.0025529365
H	-4.0881534061	2.3038241751	0.0016584579
C	-2.2816437614	1.1330762729	0.0037861918
H	-1.6017925556	1.9736044879	0.0039227313
N	-5.8932741887	0.5167786861	0.0012372202
N	-6.6432719879	-0.4722058922	0.0010604964
C	-8.0304029302	-0.1799708351	-0.0017975551
C	-8.8761189125	-1.2830313201	-0.0020161431
H	-8.4326722978	-2.2714736015	-0.0001018624
C	-10.2513660227	-1.1068865885	-0.0045869984
H	-10.9081811153	-1.9690949404	-0.0047308341
C	-10.7821246224	0.1743000038	-0.0069673321
C	-9.9355794585	1.2793742932	-0.0067649363
H	-10.3532681239	2.2798936146	-0.0086269952
C	-8.5638690845	1.1086330077	-0.0042007248
H	-7.8890171536	1.9536646793	-0.0039715362
H	-11.8570772059	0.3161292477	-0.0089779214

para-AB(4) EEEE
60

E = -1593.84299591592

H	10.9892507098	-1.9784670502	-0.0004201434
C	11.6794511491	-1.1457574613	-0.0003580897
C	13.0475612240	-1.3401168311	-0.0001418733
H	13.4476004298	-2.3478697904	-0.0000318725
C	13.9124990007	-0.2493876764	-0.0000653403
H	14.9847246360	-0.4098785979	0.0001048622
C	13.4037953214	1.0408690951	-0.0002077309
H	14.0753840656	1.8914848515	-0.0001494299
C	12.0318343803	1.2409392923	-0.0004281986
H	11.6035951367	2.2360472204	-0.0005477049
C	11.1690280806	0.1516555405	-0.0005014876
N	9.7857103160	0.4636955635	-0.0007326694
N	9.0234775700	-0.5158448425	-0.0007497359
C	7.6416981814	-0.1989137291	-0.0010099160
C	6.7738786224	-1.2897738304	-0.0010538775
H	7.2013652718	-2.2851772819	-0.0008918928
C	5.4070014540	-1.0990877809	-0.0012966073
H	4.7191464327	-1.9334241930	-0.0013380983
C	4.8976356224	0.1979578584	-0.0014972304
C	5.7640424540	1.2891560004	-0.0014530055
H	5.3346514357	2.2837059113	-0.0016143627
C	7.1309725017	1.0976414538	-0.0012126764
H	7.8180957837	1.9321620018	-0.0011758044
N	3.5166185258	0.5128221400	-0.0017660101
N	2.7542048150	-0.4675625557	-0.0018032980
C	1.3733304754	-0.1534505926	-0.0017312354
C	0.5084418610	-1.2468288207	-0.0018119196
H	0.9385566450	-2.2410670371	-0.0019147115
C	-0.8586950577	-1.0596554230	-0.0017613160
H	-1.5444646525	-1.8956064888	-0.0018259898
C	-1.3716627719	0.2359618661	-0.0016208813
C	-0.5083938221	1.3297612623	-0.0015345631
H	-0.9405727601	2.3230935269	-0.0014213407
C	0.8590522314	1.1421229686	-0.0015939119
H	1.5436066229	1.9787749347	-0.0015348352
N	-2.7545600959	0.5453434791	-0.0015505076
N	-3.5103129311	-0.4401790616	-0.0017212017
C	-4.8935194794	-0.1392894934	-0.0014185674
C	-5.7466511792	-1.2417515333	-0.0016346784
H	-5.3058487542	-2.2312936863	-0.0020089504
C	-7.1157576000	-1.0693321266	-0.0013725228
H	-7.7927292907	-1.9123992831	-0.0015322180
C	-7.6426868591	0.2202738872	-0.0008831407
C	-6.7913482999	1.3232189086	-0.0006642026
H	-7.2343575194	2.3117875604	-0.0002780918
C	-5.4217308357	1.1507095716	-0.0009323631
H	-4.7464693152	1.9949697977	-0.0007706506
N	-9.0304509479	0.5142184768	-0.0005579357

N	-9.7735939646	-0.4798678337	-0.0008581017
C	-11.1627018527	-0.1980599579	-0.0005720513
C	-11.9997305940	-1.3077691708	-0.0009002806
H	-11.5485967803	-2.2927241337	-0.0013488893
C	-13.3763081069	-1.1425865325	-0.0006489979
H	-14.0262961541	-2.0099650746	-0.0009042623
C	-13.9170869550	0.1343979546	-0.0000708001
H	-14.9931016588	0.2678655999	0.0001294370
C	-13.0792730011	1.2461353036	0.0002542505
H	-13.5048459604	2.2433153986	0.0007036937
C	-11.7062773498	1.0863092046	0.0000066103
H	-11.0381843065	1.9367035310	0.0002547139

para-AB(4) ZEEE
60
E = -1593.82115511050

H	10.4783456061	-2.0323961552	-0.8097831392
C	11.1939279389	-1.2970459170	-0.4672961018
C	12.5554599046	-1.5067604992	-0.5764573254
H	12.9245494267	-2.4277265997	-1.0135729755
C	13.4537642542	-0.5425213394	-0.1278951165
H	14.5204690580	-0.7147079432	-0.2174182374
C	12.9856004426	0.6364722342	0.4328282353
H	13.6832576393	1.3886307968	0.7824790056
C	11.6206434538	0.8511345743	0.5452236190
H	11.2229489734	1.7612203389	0.9778731121
C	10.7244099432	-0.1111661331	0.0961202174
N	9.3524470993	0.2093433979	0.2536554921
N	8.5573685581	-0.6471352502	-0.1643849145
C	7.1875872857	-0.3162316224	-0.0074053215
C	6.2805187317	-1.2463797137	-0.5125606853
H	6.6716078332	-2.1438524693	-0.9764459281
C	4.9214735813	-1.0215183371	-0.4240528795
H	4.2038871497	-1.7296680177	-0.8154515464
C	4.4604030576	0.1465224919	0.1802038252
C	5.3660518697	1.0718116138	0.6949319504
H	4.9729118029	1.9673431641	1.1607404967
C	6.7246730052	0.8480375097	0.6028176681
H	7.4414572616	1.5577573577	0.9916747061
N	3.0939752355	0.4919912674	0.3159714323
N	2.2930959227	-0.3091172430	-0.1932287609
C	0.9288338723	0.0504919126	-0.0662244431
C	0.0204486489	-0.7915153189	-0.7059980250
H	0.4063836536	-1.6534231915	-1.2367965078
C	-1.3328252679	-0.5223269242	-0.6682905940
H	-2.0502419551	-1.1588923871	-1.1676673812
C	-1.7876718361	0.5993666930	0.0222374901
C	-0.8819650582	1.4323577531	0.6759219452
H	-1.2701252779	2.2930845896	1.2069506141

C	0.4712777613	1.1655784333	0.6340841721
H	1.1880502283	1.8061081556	1.1285345307
N	-3.1471562189	0.9878960701	0.1103895782
N	-3.9422386092	0.2653664605	-0.5128399493
C	-5.2969996904	0.6683807700	-0.4462983454
C	-6.1959888729	-0.1115051088	-1.1657594139
H	-5.8144764108	-0.9653627712	-1.7122086989
C	-7.5436423893	0.1992449620	-1.1806024407
H	-8.2409615968	-0.4101979379	-1.7411704062
C	-7.9951449416	1.3108975694	-0.4778710553
C	-7.0922013374	2.1177719384	0.2131037859
H	-7.4635718747	3.0066450168	0.7094361584
C	-5.7544750266	1.7867878888	0.2525923399
H	-5.0423228459	2.3928540634	0.7953176673
N	-9.3403403153	1.7880494751	-0.5721132895
N	-10.3331524189	1.0797994125	-0.3790991558
C	-10.2471154085	-0.2605794923	0.1172595330
C	-9.5423360712	-0.5893449391	1.2703056943
H	-8.9681437262	0.1675010716	1.7895234900
C	-9.5915238293	-1.8868437096	1.7530673103
H	-9.0490862912	-2.1422427025	2.6561308150
C	-10.3249873201	-2.8575673722	1.0838904559
H	-10.3525298333	-3.8731186342	1.4618372584
C	-11.0409377831	-2.5198143975	-0.0568077962
H	-11.6297786488	-3.2700625192	-0.5720105166
C	-11.0248613681	-1.2162619229	-0.5256657690
H	-11.6062063927 -	0.9215278055	-1.3915172665

para-AB(4) EZEE
60
E = -1593.82072161510

H	-7.6399465821	-2.6611955035	1.7073550710
C	-8.3247536747	-2.7467593936	0.8747251884
C	-9.4169955757	-3.5921056679	0.9193505329
H	-9.6011384502	-4.1880595462	1.8061217636
C	-10.2806953842	-3.6830875377	-0.1688559499
H	-11.1349351452	-4.3495277913	-0.1268675679
C	-10.0504175705	-2.9231801640	-1.3058068303
H	-10.7218055649	-2.9930325926	-2.1539061113
C	-8.9562664843	-2.0733344403	-1.3554937799
H	-8.7476308995	-1.4678545909	-2.2294598981
C	-8.0935730368	-1.9842774546	-0.2696989552
N	-7.0035799337	-1.0926953163	-0.4244191563
N	-6.2313560852	-1.0321771832	0.5453720751
C	-5.1380916247	-0.1459804524	0.3810243823
C	-4.2165631906	-0.1252306881	1.4257285882
H	-4.3991523242	-0.7638179516	2.2815401146

C	-3.0990472627	0.6811232316	1.3562308104
H	-2.3651266466	0.7001026298	2.1499319942
C	-2.9032679007	1.4812920950	0.2323097597
C	-3.8374282583	1.4768749754	-0.8022268738
H	-3.6568663884	2.1149434469	-1.6589317944
C	-4.9515239483	0.6655081001	-0.7357356305
H	-5.6815131265	0.6377711403	-1.5330974942
N	-1.7771903427	2.3155439478	0.0357481795
N	-0.9094200095	2.2414863645	0.9212185790
C	0.2353913621	3.0415624041	0.6985227779
C	1.2996379405	2.8297273308	1.5667827149
H	1.1748539972	2.1132535796	2.3693109191
C	2.4996841875	3.4879967341	1.3735667793
H	3.3432618233	3.2972196772	2.0245090980
C	2.6213365198	4.3805902392	0.3158113653
C	1.5261070926	4.6732540653	-0.4933399569
H	1.6327726915	5.4195582445	-1.2718496749
C	0.3432844661	3.9848897022	-0.3234015366
H	-0.5061950886	4.1550611558	-0.9708203149
N	3.8352920820	5.0961972669	0.0721758715
N	4.8987159516	4.5017552629	-0.1377266720
C	4.9557454766	3.0765411317	-0.2713443668
C	5.9023324804	2.4045141113	0.4984538264
H	6.5192855695	2.9740460144	1.1836696900
C	6.0306419355	1.0344660482	0.3977468121
H	6.7372886902	0.4893028195	1.0085644847
C	5.2454633889	0.3319129023	-0.5160833543
C	4.3483640824	1.0156818969	-1.3277218913
H	3.7655359836	0.4482457637	-2.0430542543
C	4.1941194970	2.3842338993	-1.2067119497
H	3.4828718662	2.9093595139	-1.8309231057
N	5.2921048079	-1.0722153937	-0.7008854169
N	6.0733124769	-1.6755231051	0.0511974347
C	6.1124183677	-3.0821145524	-0.1257860100
C	6.9994485125	-3.7684178364	0.6948945097
H	7.5932859085	-3.1997520073	1.4003596326
C	7.1105834408	-5.1474827393	0.6015528164
H	7.8042061043	-5.6794964546	1.2424111852
C	6.3319770251	-5.8420508653	-0.3120636342
H	6.4153487378	-6.9204695580	-0.3874589949
C	5.4417994452	-5.1551902302	-1.1326208254
H	4.8331153450	-5.7002067278	-1.8454296616
C	5.3284209770	-3.7805071121	-1.0442886363
H	4.6418509874	-3.2298705448	-1.6729982660

para-AB(4) ZZEE
60
E = -1593.79797978619

H	-7.9549767212	-1.3435401669	2.1019344474

C	-8.6239502461	-1.6195178063	1.2982123210
C	-9.8188119057	-2.2700799088	1.5410943672
H	-10.1020520243	-2.5139402453	2.5587743277
C	-10.6593130718	-2.6145997128	0.4859710732
H	-11.5946235693	-3.1259124254	0.6846459346
C	-10.3014041703	-2.3068191603	-0.8180000191
H	-10.9539906683	-2.5752372744	-1.6407758399
C	-9.1031760500	-1.6555959956	-1.0671107188
H	-8.7936274481	-1.4037743915	-2.0744472504
C	-8.2653194518	-1.3106956531	-0.0137141937
N	-7.0627248851	-0.6552079148	-0.3765280766
N	-6.3340719713	-0.3305393111	0.5741375045
C	-5.1233638954	0.3076936612	0.2078386165
C	-4.2749549977	0.6358020171	1.2629359215
H	-4.5974891227	0.4015783485	2.2701652475
C	-3.0533082953	1.2287381594	1.0192360840
H	-2.3745003212	1.4773219587	1.8230162736
C	-2.6750122649	1.4982074786	-0.2947651612
C	-3.5321383715	1.1858190977	-1.3487017079
H	-3.2070614256	1.4088368813	-2.3578456377
C	-4.7545872629	0.5934663070	-1.1049682454
H	-5.4297947671	0.3332344806	-1.9086357674
N	-1.4235109617	2.0476611861	-0.6632629192
N	-0.6748163003	2.3242932031	0.2882268685
C	0.6057122934	2.8002216993	-0.0766100050
C	1.4973307434	2.9926654362	0.9724698368
H	1.1471618955	2.8233897545	1.9831630835
C	2.8099407734	3.3414111346	0.7174796549
H	3.5180321639	3.4513268215	1.5286724295
C	3.2237010098	3.5116162798	-0.5982358765
C	2.3078928630	3.4274475815	-1.6444297045
H	2.6455183930	3.6259177752	-2.6549861750
C	1.0074305007	3.0450780093	-1.3899633428
H	0.2903221238	2.9170148683	-2.1894827204
N	4.5740631660	3.8508985789	-0.9245452899
N	5.5189601277	3.1313161836	-0.5783720795
C	5.3100532511	1.8663449733	0.0635432947
C	4.4819746319	0.8787394548	-0.4645424871
H	3.9255289559	1.0628837506	-1.3742676785
C	4.3631031648	-0.3384067930	0.1760632977
H	3.7151481417	-1.1017496666	-0.2343424677
C	5.0535451436	-0.5692519423	1.3633326664
C	5.8739913603	0.4197633561	1.8918838742
H	6.3792862344	0.2327232468	2.8319673742
C	6.0435980766	1.6142393002	1.2151503122
H	6.7117312713	2.3800211941	1.5908350847
N	4.8414330977	-1.7308891717	2.1733432267
N	4.8598806985	-2.8834363971	1.7300380311
C	5.2566034282	-3.1945193684	0.3898351975

C	6.4757417660	-2.7824479013	-0.1371824667
H	7.1275650631	-2.1408833032	0.4425527206
C	6.8510354121	-3.2070963190	-1.4011562841
H	7.8041822974	-2.8919950822	-1.8100256741
C	6.0111980538	-4.0252255774	-2.1448746160
H	6.3060557740	-4.3467696111	-3.1371219214
C	4.8031759940	-4.4480203747	-1.6065260079
H	4.1521256612	-5.1010805037	-2.1764620661
C	4.4365743781	-4.0557421186	-0.3291890091
H	3.5155277105	-4.4031558771	0.1242535836

para-AB(4) ZZZE
60
E = -1593.77688534358

H	-6.9825250413	-0.3584433032	1.8899301588
C	-7.4070176901	-1.1980745682	1.3565577513
C	-8.3688090725	-2.0086053117	1.9294635316
H	-8.7123703309	-1.8063790667	2.9375741351
C	-8.8987545960	-3.0823229074	1.2190322954
H	-9.6535712665	-3.7127955343	1.6753568523
C	-8.4621641589	-3.3469620355	-0.0704366546
H	-8.8726991199	-4.1826836692	-0.6253585753
C	-7.4957819723	-2.5385921714	-0.6485549098
H	-7.1320103433	-2.7198872698	-1.6528929019
C	-6.9688789533	-1.4654901528	0.0600197923
N	-5.9847020535	-0.7074634231	-0.6211880678
N	-5.5376609794	0.2656166200	0.0060139340
C	-4.5423780111	1.0057705305	-0.6784539258
C	-3.9990451819	2.0807629587	0.0154262416
H	-4.3753870899	2.2950260877	1.0081201117
C	-2.9900400120	2.8403672328	-0.5478170978
H	-2.5540734616	3.6674522050	-0.0020066041
C	-2.5312382868	2.5238893393	-1.8211811770
C	-3.1223853575	1.4923524997	-2.5480503358
H	-2.7828800340	1.3049275741	-3.5600316794
C	-4.1061620809	0.7173466275	-1.9717035020
H	-4.5564469644	-0.1093048404	-2.5041238795
N	-1.5370415163	3.3002555499	-2.4920987997
N	-0.4298833153	3.5376693228	-1.9971961465
C	0.0177073021	2.9501507386	-0.7703307842
C	-0.0749304896	1.5882165127	-0.4911317556
H	-0.5721844412	0.9213719318	-1.1829528060
C	0.4800448518	1.0831002001	0.6672170972
H	0.4095116775	0.0243312747	0.8781002997
C	1.1192839086	1.9372899668	1.5635146391
C	1.1833799448	3.2995964433	1.2982872901
H	1.6520154981	3.9518431458	2.0252437822
C	0.6787600889	3.7964798868	0.1103490886
H	0.7686136638	4.8459711886	-0.1427923617

N	1.6208433612	1.5140222066	2.8351546226
N	2.3180427085	0.5049891524	2.9873552778
C	2.8147385356	-0.2719420947	1.8937219359
C	3.5288398748	0.2817076183	0.8344477894
H	3.6688847350	1.3535045497	0.7750219226
C	4.0613192567	-0.5391694487	-0.1405144502
H	4.6219020120	-0.1099225788	-0.9606098057
C	3.8608658395	-1.9160984583	-0.0760483662
C	3.1257848345	-2.4631596708	0.9684829680
H	2.9505629539	-3.5324000005	0.9811777232
C	2.6505389579	-1.6490088686	1.9797089828
H	2.1195731254	-2.0629483377	2.8287363828
N	4.2506722286	-2.8150334992	-1.1209166877
N	5.3824354718	-2.8284866818	-1.6158147260
C	6.4619662211	-2.0423689462	-1.0979744556
C	6.8505179297	-2.0953168428	0.2362093914
H	6.2900585307	-2.6976711402	0.9402806139
C	7.9586744079	-1.3761835885	0.6518232753
H	8.2649736246	-1.4188553948	1.6907124140
C	8.6693057946	-0.5976988579	-0.2524465488
H	9.5291666459	-0.0282257548	0.0805932352
C	8.2886906968	-0.5665499370	-1.5874013660
H	8.8508979218	0.0260827070	-2.2999271729
C	7.2014891034	-1.3100461030	-2.0173964557
H	6.9059853853	-1.3275691294	-3.0600203062

para-AB(4) ZZZZ
60
E = -1593.75485608512

H	7.1078511949	-1.5929479191	-0.7296505996
C	7.4897962126	-0.6615206381	-0.3309625300
C	8.2603981874	-0.6554497784	0.8198832318
H	8.4790638160	-1.5914478930	1.3210853814
C	8.7460624830	0.5397806861	1.3333090200
H	9.3418904311	0.5386308927	2.2385769823
C	8.4863666412	1.7325522358	0.6716361471
H	8.8797397595	2.6658979943	1.0575045097
C	7.7447263364	1.7301461242	-0.4984562766
H	7.5626637406	2.6429216957	-1.0536342091
C	7.2170626466	0.5383147548	-0.9794202460
N	6.5260179235	0.6045772610	-2.2328637475
N	5.4372800646	0.0567389723	-2.4333040005
C	4.6764781235	-0.5813183111	-1.4022578088
C	4.3593459439	0.0405093452	-0.1972126400
H	4.7715635153	1.0136619409	0.0349190990
C	3.5090903633	-0.5816613488	0.6967748907
H	3.2526560890	-0.0960480881	1.6294579279
C	2.9940411693	-1.8410999209	0.4016392859
C	3.3358727015	-2.4744718527	-0.7870736173

H	2.9484697451	-3.4673241264	-0.9828544268
C	4.1313913204	-1.8223079814	-1.7105309760
H	4.3592934336	-2.2710409555	-2.6701294147
N	2.2150673642	-2.5982829176	1.3326201508
N	1.2183670178	-2.1452845351	1.9048345017
C	0.6426237139	-0.8738260116	1.5883463376
C	0.3193159638	-0.4901689697	0.2894102818
H	0.5845496506	-1.1232432998	-0.5474616974
C	-0.3600677820	0.6919916467	0.0697600269
H	-0.6223925549	0.9835515949	-0.9390329277
C	-0.6993733835	1.5098062886	1.1443850261
C	-0.3446603398	1.1433629022	2.4367261427
H	-0.5883608962	1.8083705758	3.2566303239
C	0.2765037197	-0.0714998440	2.6619773630
H	0.5041480563	-0.4068309709	3.6667592857
N	-1.3005180186	2.7974942862	0.9754631171
N	-2.3111883097	2.9798704026	0.2879770304
C	-3.0637537861	1.9046770026	-0.2827146947
C	-3.5482079582	0.8421719202	0.4751970837
H	-3.2962431188	0.7649454824	1.5249797572
C	-4.3593850674	-0.1102491879	-0.1113056563
H	-4.7440413610	-0.9318821959	0.4791117612
C	-4.6649602104	-0.0219381638	-1.4667459251
C	-4.1603707509	1.0258557959	-2.2269231371
H	-4.3824887498	1.0582951212	-3.2870285293
C	-3.4076022013	2.0166975154	-1.6244781729
H	-3.0502990512	2.8691023652	-2.1898930964
N	-5.3621508256	-1.0538646743	-2.1730368070
N	-6.4471328734	-1.5144398690	-1.8046088897
C	-7.1987476745	-0.9637062738	-0.7165330113
C	-7.5188870353	0.3874835328	-0.6314093573
H	-7.1319985847	1.0840223739	-1.3644830980
C	-8.3440441168	0.8300084904	0.3892835238
H	-8.5995922573	1.8814777850	0.4524623049
C	-8.8371497351	-0.0641751124	1.3303639487
H	-9.4750402752	0.2890666935	2.1323266329
C	-8.5313303862	-1.4146707839	1.2283934494
H	-8.9310542049	-2.1189877115	1.9490208913
C	-7.7349278395	-1.8697141440	0.1901659217
H	-7.5158149021	-2.9239584231	0.0672238406
